# Synthesis and characterization of mesoporous silica supported metallosalphen-azobenzene complexes: efficient photochromic heterogeneous catalysts for the oxidation of cyclohexane to produce KA oil

**DOI:** 10.1039/d4ra04698f

**Published:** 2024-08-27

**Authors:** Salimah Alshehri, Mohamed Abboud

**Affiliations:** a Catalysis Research Group (CRG), Department of Chemistry, College of Science, King Khalid University Abha 61413 Saudi Arabia mabboud@kku.edu.sa +966 53 48 46 782

## Abstract

The oxidation of cyclohexane to produce KA oil (cyclohexanone and cyclohexanol) is important industrially but faces challenges such as low cyclohexane conversion at high KA oil selectivity, and difficult catalyst recyclability. This work reports the synthesis and evaluation of new heterogeneous catalysts consisting of Co(ii), Mn(ii), Ni(ii) and Cu(ii) salphen-azobenzene complexes [ML_1_] immobilized on amino-functionalized mesoporous silica (SBA-15, MCM-41, MCM-48) through coordination bonding. In the first step, the salphen-azobenzene ligand was synthesized and complexed with Co, Mn, Ni and Cu metal ions. In the second step, aminopropyltriethoxysilane (APTES) was grafted onto the surface of different types of commercial mesoporous silica. The immobilization of [ML_1_] onto the mesoporous silica surface and the thermal stability of the obtained materials were confirmed using different characterization techniques such as FT-IR, powder XRD, SEM, TEM, BET, and TGA. The obtained results revealed high dispersion of [ML_1_] through the silica surface. The catalytic activity of the prepared materials Silica-N-ML_1_ was evaluated on the cyclohexane oxidation to produce KA oil using various oxidants. The *cis*–*trans* isomerization of the azobenzene upon UV irradiation was found to affect the catalytic performance of Silica-N-ML_1_. The *cis* isomer of SBA-15-N-CoL_1_ exhibited the highest cyclohexane conversion (93%) and KA selectivity (92%) under mild conditions (60 °C, 6 h) using *m*-CPBA as oxidant. Moreover, The SBA-15-N-CoL_1_ showed high stability during four successive cycles.

## Abstract 2

The oxidation of cyclohexane to produce KA oil (cyclohexanone and cyclohexanol) is important industrially but faces challenges such as low cyclohexane conversion at high KA oil selectivity, and difficult catalyst recyclability.

## Graphical abstract

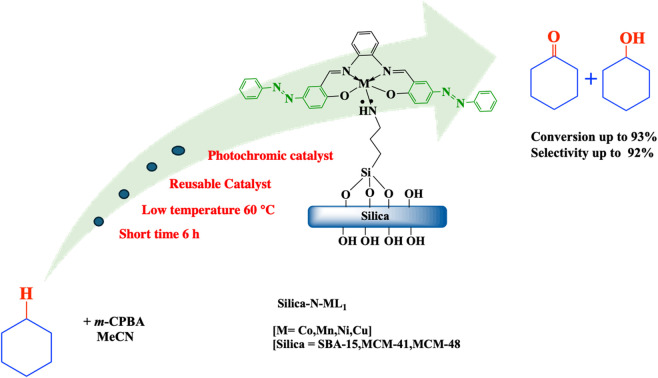

## 1. Introduction

**Scheme 1:**
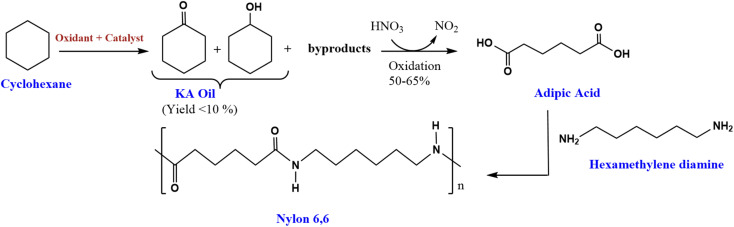
Synthesis route of nylon 6,6 from cyclohexane.^2^

The oxidation of cyclohexane is an important industrial chemical reaction. This transformation produces cyclohexanol (A) and cyclohexanone (K), commonly referred to together as ketone-alcohol (KA) oil.^1^ KA oil serves as a critical feedstock in the manufacture of nylon 6,6 fibers (Scheme 1).^2^ The production of nylon 6,6 involves further oxidation of KA oil with nitric acid to form adipic acid. Adipic acid then acts as an important building block monomer in the preparation of nylon 6,6.^3^ This nylon polymer finds extensive application in textiles due to their desirable mechanical properties.^4,5^ Additionally, adipic acid itself is an important intermediate chemical in various industrial processes. It is commonly used to manufacture other polymeric materials, resins, polyesters, and plasticizers.^5–8^

The current industrial process for the oxidation of cyclohexane to produce KA oil involves cobalt or manganese salt as homogenous catalysts under high-temperature (150–160 °C) and high-pressure conditions (10–20 atm) of air or oxygen gas.^9^ However, cyclohexane is quite stable under these conditions while the desired products cyclohexanol and cyclohexanone are less stable, resulting in numerous undesirable by-products forming at the elevated temperatures and pressures.^10^ Another limitation of this industrial process is that the conversion of cyclohexane must be kept at less than 10% to ensure high selectivity toward KA oil (around 80%).^11^ Additionally, this process also faces challenges in regenerating and reusing the homogeneous catalysts.^12^ Therefore, many efforts have been made to develop more efficient catalysts to covert cyclohexane to KA with higher conversion and selectivity under mild conditions.^13^

Over the past decades, extensive research efforts have been focused on developing heterogeneous catalysts as alternatives to address issues such as low conversion, selectivity, and the high-cost.^14,15^ In addition, heterogeneous catalysis provides important advantages over homogeneous conditions, including improved catalyst activity, easy separation from reaction mixtures, and reuse over multiple cycles.^16–20^

Various transition metal-based systems have shown promise as catalysts, including transition metal ions, organotransition metal complexes (OTMCs), and transition metal-oxo and -peroxo complexes.^21–23^ Some OTMCs have shown more promise as effective catalysts for the oxidation of cyclohexane.^24^ Notably, the most extensively investigated OTMCs for cyclohexane oxidation are Schiff base and metallo-porphyrins metal complexes.^22,25^ Using common oxidizing agents such as hydrogen peroxide (H_2_O_2_), oxygen gas (O_2_), *tert*-butyl hydroperoxide (*t*-BuOOH), and *meta*-chloroperbenzoic acid (*m*-CPBA).^26–29^

Salphen, one of the Schiff base ligands, it has proven to be a promising ligand for OTMCs used as catalyst in the oxidation of hydrocarbons, because of its simple synthesis and structural tunability. Salphen are organic compounds with azomethine (–C=N–) groups.^30^ They are often made by condensing carbonyl compounds with *o*-phenylenediamine. The introduction of various metal centres, functional groups, and substituents is made possible by their facile synthesis and structural diversity. This enabled the performance of the obtained catalysts to be optimized for certain reactions.^31^ Transition metals such as cobalt, copper, manganese, and nickel are of more interest due to their efficiency in the oxidation of hydrocarbons, relative abundance, and lower cost, compared to precious metals such as silver and gold.^32^

Modification of metal–ligand combinations aims to develop sustainable oxidation catalysts. One promising strategy is to insert photoactive azo group into metal–ligand complexes to impart light-responsive properties.^33^ Azobenzene derivatives have many useful applications. Specifically, the *cis*/*trans* photoisomerization of azobenzene moiety can find important utilizations in optics, photochemistry, and biomaterials.^34^ Inserting such photochromic moiety into salphen ligand scaffold could impart new light-responsive functionality to the resulting OTMC catalyst.^35,36^ However, the effect of *cis*/*trans* photoisomerization of the azobenzene on the catalytic activity remains unexplored. Moreover, only few studies have investigated salphen-azobenzene-based OTMCs as catalysts in the oxidation reaction of hydrocarbons.^37^ Salem and coworkers are the most reported the synthesized and characterization of salphen-azobenzene complexes.^35^ However, on the best of our knowledge, this type of OTMCs have never been successfully employed as heterogeneous catalysts.^27^

Various solid supports have been reported for the heterogenization of salphen-based OTMCs. This is including mesoporous silicas, porous carbons, zeolites, polymers, clays and resins.^38^ Mesoporous silica materials (silica) such as MCM-41, MCM-48 and SBA-15 are considered more efficient as support due to their high surface areas and large pore sizes, narrow pore size distribution, easy functionalization, highly ordered nanostructure, different pores-network dimension (1D, 2D, and 3D), which allow easy diffusion of reactants and products, without the need for swelling agents.^39–43^

Different techniques have been used to immobilize OTMCs in silica, either onto surface or into framework, such as physical adsorption, grafting, co-condensation, periodic mesoporous organosilica (PMOs).^44,45^ The physical adsorption of OMTCs into silica surface is the easiest technique. However, due to the very weak physical bonds between the catalyst and support, this technique suffers from a major problem, which is the rapid leaching of the catalyst from the support. Previous studies have investigated immobilizing some metal–salphen complexes on solid supports *via* physical adsorption. However, challenges remained, such as leaching, lower activity, selectivity and recyclability.^44–47^ While the immobilization of OTMCs in silica through chemical bonding, including grafting, co-condensation, PMOs methods, affords more stable OTMCs@silica heterogeneous catalyst.^46,47^ However, these methods are usually more complicated than the physical approach.

Therefore, developing an efficient, stable, and reusable heterogeneous catalyst *via* a simple immobilization method, under mild conditions, remains the main object of many researchers in the field of catalysis.^48–50^

Herein, we report the synthesis, characterization, and catalytic activity evaluation of silica supported Co(ii), Mn(ii), (Ni(ii) and Cu(ii) metallosalphen-azobenzene derivatives [ML_1_]. [ML_1_] were immobilized onto silica surface *via* coordination-assisted grafting method. In this approach, 3-aminopropyltriethoxysilane (APTES) was first grafted onto the surface of different type of silica (*i.e.*, SBA-15, MCM-41, and MCM-48) to afford amino-functionalized silica (*i.e.*, SBA-15-N, MCM-41-N, and MCM-48-N). Then [ML_1_] were added to Silica-NH_2_ to form stable Silica-N-ML_1_ materials *via* coordinate bond between NH_2_ group of APTES and the metal ions (*e.g.*, SBA-15-NH_2_-Co(ii) salphen-azobenzene). The obtained nanocatalysts Silica-N-ML_1_ were characterized by TEM, SEM, FT-IR, BET, TGA and XRD. The catalytic activity of these new nanocatalysts were evaluated in the oxidation of cyclohexane using different oxidants such as *meta*-chloroperoxybenzoic acid (*m*-CPBA), *tert*-butyl hydroperoxide (*t*BuOOH, TBHP), hydrogen peroxide (H_2_O_2)_.

## 2. Experimental section

### 2.1 Materials

Hydrochloric acid (99.8%), sodium nitrite (NaNO_2_) (99.99%), sodium hydroxide (NaOH) (≥98%), salicylaldehyde (99%), aniline (99.5%), *o*-phenylenediamine (99.5%), and glacial acetic acid (99.9%) were used as the starting materials for the preparation of ligands H_2_L. The metal salts employed were cobalt(ii) acetate tetrahydrate Co(CO_2_CH_3_)_2_·4H_2_O) (≥98%), manganese(ii)acetate tetrahydrate Mn(CO_2_CH_3_)_2_·4H_2_O) (≥99%), nickel(ii) acetate tetrahydrate Ni(CO_2_CH_3_)_2_·4H_2_O) (≥99%), copper(ii) acetate tetrahydrate Cu(CO_2_CH_3_)_2_·4H_2_O) (≥98%), 3-aminopropyltriethoxysilane (APTES), commercial silica (SBA-15, MCM-41, MCM-48) cyclohexane (99%), cyclohexanone (99%), cyclohexanol (99%), chlorobenzene (99%), *meta*-chloroperoxybenzoic acid (*m*-CPBA), *tert*-butyl hydroperoxide (*t*BuOOH, TBHP), hydrogen peroxide 30% (H_2_O_2_), dichloromethane (99.8%), acetonitrile (99.9%), ethanol absolute (99.8%), chloroform (99.9%), diethyl ether (99.9%), ether (99.9%), all were purchased from Sigma Aldrich. All reagents were of analytical grade and used without further purification.

### 2.2. Methods

Proton nuclear magnetic resonance spectrum (^1^H-NMR) and (^13^C NMR) of the salphen ligand were acquired in DMSO-d^6^ solution using a Brucker AMx 600 MHz spectrometer. Elemental analysis and ICP-mass were used to determine the composition of metallosalphen-azobenzene complexes and the metal content, respectively. The UV/vis spectra of the free salphen ligand and complexation were recorded on a Shimadzu UV-1600 UV/vis spectrophotometer in the wavelength range of 250–700 nm. The morphology of the immobilized catalysts was identified by scanning electron microscopy (SEM; JSM-7100F) (JEOL (Germany) GmbH). Transmission electron microscopy (TEM) micrographs were obtained using an FEI Tecnai G2 F30 TEM operating at 200 kV using a CCD camera. TEM samples were prepared by suspending the material in ethanol using bath sonication for a few minutes, then adding a drop of the resulting suspension solution on carbon coated copper grids with lacey carbon (Ted Pella Inc.) and then letting it dry at room temperature. Important functional groups of salphen ligand complexes and immobilized systems were determined using an FTIR spectrometer (Bruker Vector 22, Ettlingen, Germany) with a wavelength range of 4000 to 500 cm^−1^. A Shimadzu Lab-XRD-6000 with CuKα radiation and a secondary monochromator was applied to measure the X-ray diffraction pattern. The thermal stability of the immobilized material was demonstrated under air using a STARe System thermogravimetric analyzer (TGA) operating at a rate of 30 mL min^−1^ from 25 to 900 °C. The specific surface area, pore volume, pore size, and pore-size distribution of immobilized samples were identified *via* using a Micrometrics ASAP 2010 apparatus (Norcross, GA). The oxidation reaction was monitored using a Shimadzu GC-2014 gas chromatography (GC) instrument equipped with a flame ionization detector and an FFAP15%CW60/80 column that was 4.0 m long, 0.32 mm in diameter and had a 1 mm film thickness. Nitrogen was used as the carrier gas at a flow rate of 35 mL min^−1^. Samples were withdrawn from the reaction mixture. The injection volume was 1 μL and the total flow rate was 35 mL min^−1^. The oven temperature was initially held at 120 °C for 1 minute then increased to 150 °C at 20 °C min^−1^ and held for 10 minutes. It was then increased to 280 °C at 50 °C min^−1^ and held for 4 minutes. The injector temperature was 190 °C and the detector temperature was 300 °C.

#### 2.2.1 Measurement of Co, Mn, Ni, and Cu content (%) using ICP-MASS

The metal content in the solid catalysts was determined using inductively coupled plasma mass spectrometry (ICP-MS). The dried catalysts were placed in digestion vessels and treated with a 4 : 1 mixture of hydrochloric acid and nitric acid. The vessels were heated to completely dissolve the catalyst matrices. The resultant digests were then filtered before analysis by ICP-MASS. The ICP-MS was first calibrated using metal standard solutions to correlate elemental emission intensities to concentration.

### 2.3 Synthesis

#### 2.3.1 Synthesis of salphen azobenzene ligand *N*,*N*′-bis[4-(benzeneazo)-2-hydroxy-3-methoxybenzaldehyde]-1,2-phenylene-diamine H_2_L_1_

**Scheme 2:**
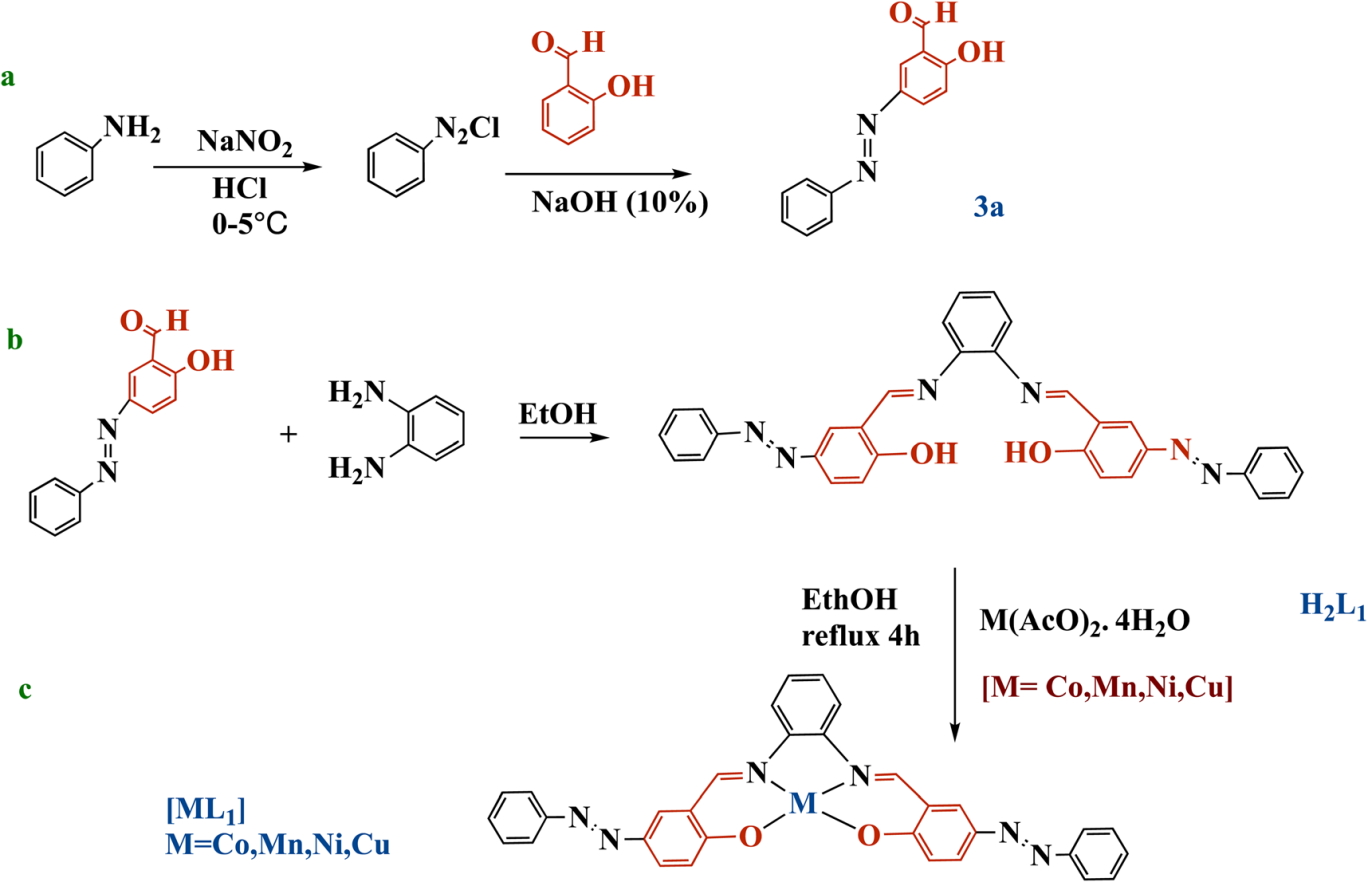
Synthesis of azobenzene precursor **3a** (a), salphen-azobenzene ligand H_2_L_1_ (b), and metal complexes [ML_1_] (M: Co, Mn, Ni, or Cu) (c).

The salphen azobenzene ligand *N*,*N*′-bis[4-(benzeneazo)salicylaldehyde]-1,2-phenylene-diamine H_2_L_1_ was synthesized according to the method reported by Sheykhi-Estalkhjani *et al.*^51^ As outlined in Scheme 2a, the synthesis first involved the preparation of the azobenzenic intermediate (3a). Aniline (0.10 mol) was dissolved in an aqueous solution of hydrochloric acid to form solution I. In a separate flask, sodium nitrite (0.12 mol) was dissolved in deionized water at 0 °C to yield solution II. Solution II was then added dropwise to solution I at 0 °C, and the resulting diazonium salt mixture was stirred for 30 minutes. Concurrently, salicylaldehyde (0.10 mol) was dissolved in a 10% sodium hydroxide solution (0.10 mol) in another flask maintained at 0 °C. The diazonium salt solution was slowly added to the salicylaldehyde solution. Stirring at 0 °C for 2 h resulted in the precipitation of an orange solid. This precipitate was collected *via* filtration, dried overnight, and named as compound (3a). Yield: 95%; mp: 185 °C; FT-IR: *ν*(C=O) 1675, *ν*(N=N) 1471, *ν*(C=C) 1463, *ν*(O–H) 3447, *ν*(C–H, aromatic), 2963. ^1^H NMR (DMSO-d^6^ as solvent, *δ* ppm): *δ* 13 (s, 1H, O–H), *δ* 10.3 (s, 1H, CHO), 8.52–7.95 (m, 8H, aromatic C–H). ^13^C NMR (DMSO-d^6^, ppm): 193 (CHO), 163.98 (C–OH), 153 (=N–C), 152 (C–N=), 115–153 (aromatic C atoms), (*k*_max_, nm): UV-Vis (CHCl_3_) (*k*_max_, nm): 330, 441.

Ligand H_2_L_1_ was synthesized through the condensation between (0.04 mol) of obtained salicylaldehyde azobenzene (3a) and (0.02 mmol) of *o*-phenylenediamine, using ethanol as solvent (Scheme 2b). The reaction was stirred and refluxed for 6 h. Then the dark yellowish brown precipitated product was cooled down at room temperature, then filtered, rinsed with ethanol, and recrystallized in ethanol. The obtained orange crystals were also filtered, and then dried in air. Yield: 82%; mp: 250 °C; FT-IR *ν*(C=N) 1606, *ν*(N=N) 1446, 1284, *ν*(C=C) 1463, *ν*(OH) 3447. ^1^H NMR (DMSO-d^6^ as solvent, *δ* ppm): 13.71 (s, 2H, O–H); 8.99 (s, 2H, CH=N), 7.95–7.06 (m, 20 H, aromatic C–H). ^13^C NMR (DMSO-d^6^), (*δ* ppm):163.98 (C–OH): 160 (C=N), 163.98, 118.01–150.89 (aromatic C atoms). UV-Vis (CHCl_3_) (*k*_max_, nm): 357, 456.

#### 2.3.2 Synthesis of metallosalphen-azobenzene complexes [ML_1_]

All complexation prepared following Salem *et al.* procedure as described below.^35^

##### 2.3.2.1 Synthesis of [CoL_1_]

(0.012 mol) of H_2_L_1_ was first dissolved in 50 mL of ethanol. Then (0.012 mol) of cobalt(ii) acetate tetrahydrate solution was added dropwise to H_2_L_1_ solution, and the mixture was refluxed for 4 h. The complex was isolated from mixture as the dark brown by filtration, then washed with ethanol and ether. Drying in air yielded [CoL_1_] complex as a dark brown solid. Yield: 70%; mp: 273 °C; FT-IR: *ν*(C=N) 1592, *ν*(N=N)1435, *ν*(C–O) 1287, *ν*(C=C) 1520, *ν*(Co–O) 678, *ν*(Co–N) 499. UV-Vis (CHCl_3_) (*k*_max_, nm): 381, 460, and 467. Elemental and ICP-MS analysis for compound (C_32_H_22_CoN_6_O_2_) MW (581.50 g mol^−1^): calculated: C, 66.10%; H, 3.81%; Co, 10.13%; N, 14.45%; O, 5.50%, found C, 67.10; H, 4.51; N, 15.05; O, 6.50 Co, 7.4%

##### 2.3.2.2 Synthesis of [MnL_1_]

(0.012 mol) of H_2_L_1_ was first dissolved in 50 mL of ethanol. Then (0.012 mol) of manganese(ii) acetate tetrahydrate solution was added dropwise to H_2_L_1_ solution, and the mixture was refluxed for 4 h. The complex [MnL_1_] was isolated from mixture as a deep brown solid by filtration, then washed with ethanol and ether. Yield: 69% C; FT-IR *ν*(C=N) 1597, *ν*(N=N) 1430, *ν*(C–O) 1287, *ν*(C=C) 1520, *ν*(Mn–O) 774, (Mn–N) 506; UV-Vis (CHCl_3_) (*k*_max_, nm): 400, 462. Elemental and ICP-MS analysis for compound (C_32_H_22_Mn N_6_O_2_) MW (577.51 g mol^−1^) calculated: C, 66.55%; H, 3.84%; Mn, 9.51%; N, 14.55%; O, 5.54%, found C, 65.99%; H, 4.96%; N, 14.10%; O, 10.20%; Mn, 8.2%.

##### 2.3.2.3 Synthesis of [NiL_1_]

(0.012 mol) of H_2_L_1_ was dissolved in 50 mL of ethanol. (0.012 mol) of nickel(ii) acetate tetrahydrate solution was added dropwise to the ligand solution and refluxed for 4 hours. After refluxing, the solution had turned dark red. The complex was isolated by filtration and purified by washing with ethanol and ether. Drying yielded [NiL_1_] as a dark red solid. Yield: 72%; mp: 274 °C; FT-IR: *ν*(C=N) 1594, *ν*(N=N) 1432, *ν*(C–O) 1284, *ν*(C=C) 1517, *ν*(Ni–O) 667, *ν*(Ni–N) 495. UV-Vis (CHCl_3_) (*k*_max_, nm): 381, 460, and 467. Elemental and ICP-MS analysis for compound (C_32_H_22_N_6_NiO_2_) MW (581.26 g mol^−1^): calculated: C, 66.12%; H, 3.82%; N, 14.46%; Ni, 10.10%; O, 5.50%, found C, 65.99%; H, 4.96%; N, 15.3%; O, 10.28%; Ni, 9.1%

##### 2.3.2.4 Synthesis of [CuL_1_]

The ligand H_2_L_1_ (0.012 mol) was dissolved in ethanol. A solution of copper(ii) acetate tetrahydrate (0.012 mol) was added dropwise to the ligand solution. The mixture was refluxed for 4 hours. During refluxing, the solution turned dark yellowish brown, indicating the formation of the complex. The complex was isolated from mixture as a dark yellowish brown solid by filtration, then washed with ethanol and ether. Yield: 70%; mp: 276 °C; FT-IR: *ν*(C=N)1590, *ν*(N=N)1430, *ν*(C–O) 1285, *ν*(C=C) 1518, *ν*(Cu–O) 663, *ν*(Cu–N) 492. UV-Vis (CHCl_3_) (*k*_max_, nm): 380, 457, and 467. Elemental and ICP-MS analysis for compound (C_32_H_22_CuN_6_O_2_) MW (586.11 g mol^−1^): calculated: C, 65.58%; H, 3.78%; Cu, 10.84%; N, 14.34%; O, 5.46%, found C, 65.1%; H, 4.98%; N, 15.3%; O, 10.88%; Co, 8.1%

#### 2.3.3 Synthesis of amino-functionalized silica: SBA-15-NH_2_, MCM-41-NH_2_, and MCM-48-NH_2_

Amino-functionalized silica were prepared *via* a post-synthesis grafting method based on the procedure reported by Abboud *et al.*^52^ The synthesis involved dispersing the commercial silica (*i.e.*, SBA-15, MCM-41, or MCM-48) in toluene, followed by dropwise addition of 3-aminopropyltriethoxysilane (APTES). Specifically, in 1 L two-neck round-bottom flask equipped with a magnetic stirrer and a reflux condenser, 5 g of commercial silica (SBA-15, MCM-41, or MCM-48) was dispersed in 650 mL of toluene, then 5.8 mL (25 mmol) of APTES was added dropwise to silica. After agitating the mixture for 15 h at 70 °C, the obtained product was separated by filtration, washed thoroughly with toluene and ethanol (200 mL, 3 times) to remove the excess of APTES. The obtained solid was then dried 2 days at 100 °C. The obtained materials were named as SBA-15-N, MCM-41-N, and MCM-48-N.

#### 2.3.4 Immobilization of metallosalphen-azobenzene complexes [ML_1_] onto SBA-15-N, MCM-41-N, and MCM-48-N surface

**Scheme 3:**
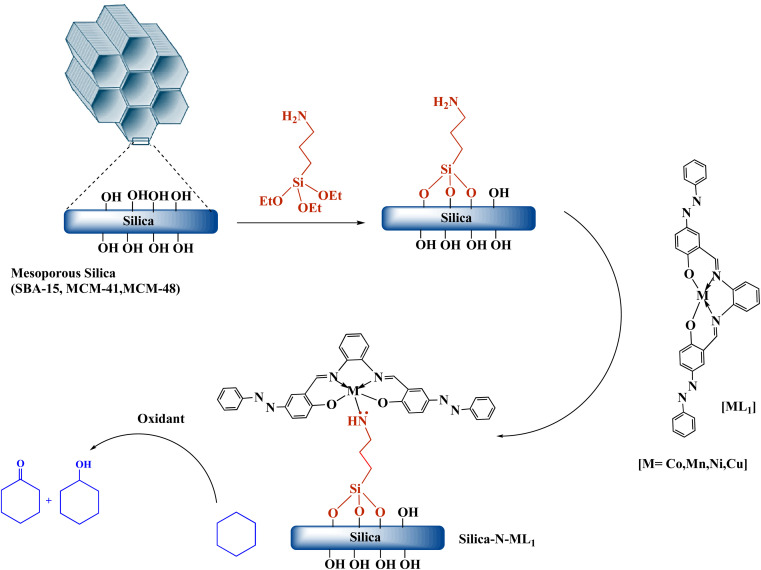
Synthesis of the nanocatalysts Silica-N-ML_1_ (Silica: SBA-15, MCM-41 or MCM-48; M: Co, Mn, Ni or Cu).

The immobilization of metallosalphen-azobenzene complexes [ML_1_] (*i.e.*, CoL_1_, MnL_1_, NiL_1_ and CuL_1_) into amino-functionalized silica Silica-NH_2_ (*i.e.*, SBA-15-N, MCM-41-N, and MCM-48-N) was carried out following a procedure reported by Abboud *et al.* (Scheme 3).^52^ Briefly, in a 250 mL flask, 1 g of Silica-NH_2_ was dispersed in 100 mL of EtOH/CH_2_Cl_2_ (2 : 1 v/v). Then 0.5 g of [ML_1_] was added to the mixture. The desired catalysts Silica-N-ML_1_ (Silica: SBA-15, MCM-41 or MCM-48, M: Co, Mn, Ni or Cu) were obtained after stirring the mixture at 50 °C for 15 h, followed by filtration and washing thoroughly with CH_2_Cl_2_ and EtOH to remove the excess of [ML_1_], and drying the obtained solid overnight at 80 °C for 15 h.

#### 2.3.5 Oxidation of cyclohexane

The oxidation of cyclohexane was performed following a procedure reported by Arumugam *et al.*^53^ Briefly, 100 mg of the catalyst dispersed in 5 mL of acetonitrile as solvent, 1.0 mL (10 mmol) of CXAN, 1.0 mL (10 mmol) of chlorobenzene as internal standard, and a specific amount of oxidant were added in a autoclave reactor, then the reactor was closed and heated at 60 °C for 6 h. Different oxygen donors were evaluated under these conditions, including hydrogen peroxide (H_2_O_2_), *tert*-butyl hydroperoxide (TBHP), and *meta*-chloroperoxybenzoic acid (*m*-CPBA).

The conversion of cyclohexane and selectivity of cyclohexanone and cyclohexanol were calculated according to the following equations:
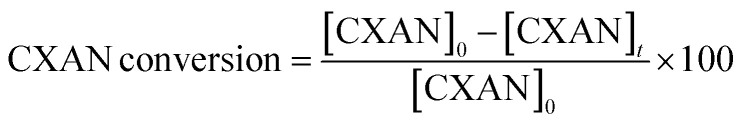
where [CXAN]_0_ is the initial cyclohexane concentration at time = 0, [CXAN]_*t*_ is the concentration of cyclohexane after 6 hours of the reaction.
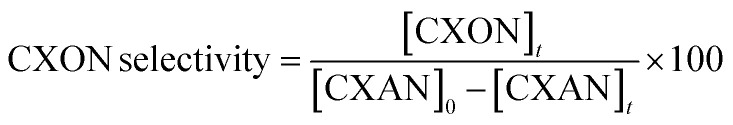
where [CXON]_*t*_ is the cyclohexanone concentration after 6 hours
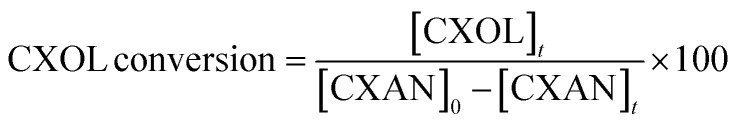
where [CXOL]_*t*_ is the cyclohexanone concentration after 6 hours.

#### 2.3.6 Effect of *cis*/*trans* conformation of the catalyst

This study examined the *cis*/*trans* isomerization of azobenzene in the catalyst SBA-15-N-CoL_1_. A solution of SBA-15-N-CoL_1_ (100 mg) in acetonitrile was placed in an autoclave and irradiated for 45 min with UV light 365 nm. Then, 1.0 mL (10 mmol) of cyclohexane, 1.0 mL (10 mmol) of chlorobenzene, and 2.50 g of *m*-CBPA were added to the autoclave. The autoclave was sealed, and heated for 6 h at 60 °C.

#### 2.3.7 Catalyst reuse and stability

The recyclability of SBA-15-N-CoL_1_ catalyst was tested over multiple runs. After each cycle, the catalyst was separated from the reaction mixture by filtration, then dispersed in chloroform for 30 minutes, washing away bound materials. Then the catalyst was isolated by centrifuge from the chloroform. Drying at 70 °C overnight prepared the catalyst for the next cycle under the same conditions.

## 3. Results and discussion

### 3.1 Synthesis and characterization

**Fig. 1:**
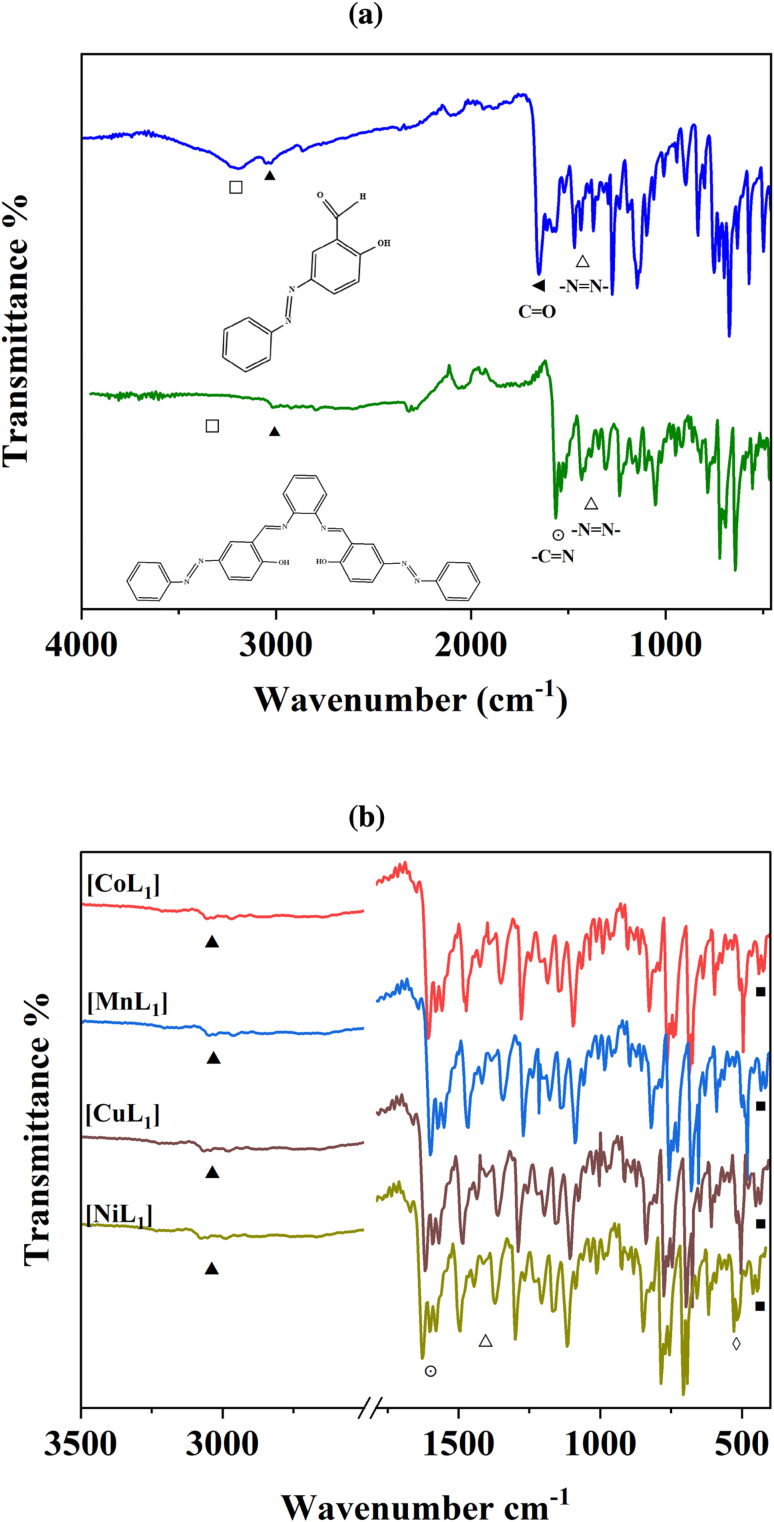
FTIR spectra of (3a) and H_2_L_1_ (a), and its corresponding Co, Mn, Ni, and Cu complexes (b).

FT-IR spectroscopy was used to characterize the structures of the synthesized salphen-azobenzene ligand H_2_L_1_ and its Co, Mn, Ni, and Cu complexes. The IR spectrum of H_2_L_1_ (Fig. 1a) confirmed the formation of the salphen backbone, due the disappearance of the aldehyde carbonyl peak at 1703 cm^−1^ (◄) of the azobenzene compound (3a), and appearance of a new imine stretching band at 1606 cm^−1^ (⊙). Additional characteristic peaks were observed at 1446 cm^−1^ (Δ) assigned to the N=N stretching, and 3200 cm^−1^ (▲) corresponding to aromatic C–H stretching. Furthermore, the broad peak at 3447 cm^−1^ (□) was assigned to O–H stretching.

The coordination of Co(ii), Mn(ii), Ni(ii), and Cu(ii) to H_2_L_1_ was indicated by shifts in the C=N stretching bands to lower wavenumbers of 13–25 cm^−1^ (⊙) in the spectra of [CoL_1_], [MnL_1_], [NiL_1_], and [CuL_1_] compared to H_2_L_1_ (Fig. 1b). The O–H stretching in H_2_L_1_ is absent in the complexes, confirming the deprotonation and coordination of O–H to the metal centers. Finally, new weak bands in the low wavenumber region of 678–674 cm^−1^ (◊) and 499–506 cm^−1^ (■) provide evidence of M–O and M–N bonding in the Co, Mn, Ni, and Cu complexes. Overall, the FT-IR analyses indicated the successful synthesis of the salphen-azobenzene ligand and its Co(ii), Mn(ii), Ni(ii), and Cu(ii) metal complexes.

**Fig. 2:**
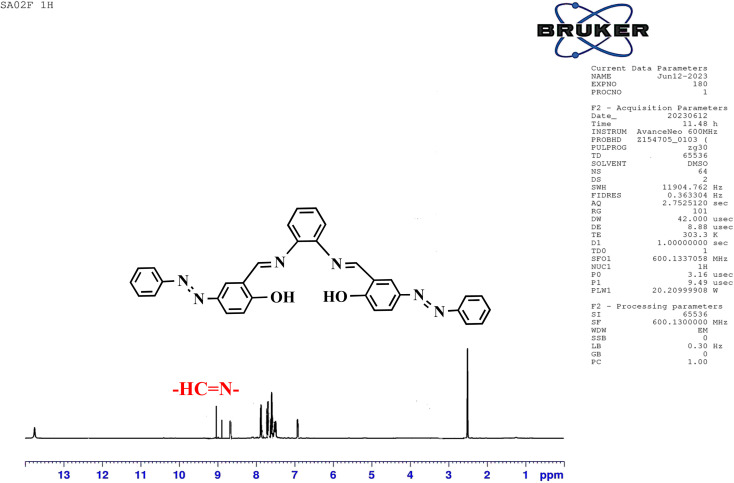
^1^H NMR spectra of H_2_L_1_.

**Fig. 3:**
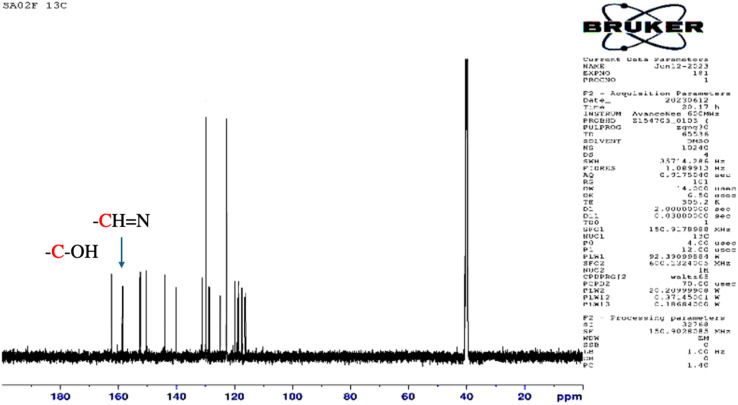
^13^C NMR spectra of H_2_L_1_.

The structure of the synthesized salphen-azobenzene ligand H_2_L_1_ was confirmed by the ^1^H and ^13^C NMR analysis. As shown in Fig. 2, the ^1^H NMR spectrum displays a singlet at *δ* 13.23 ppm assigned to the phenolic –OH protons, indicating an enolic contribute to the structure as reported previously.^54^ Additionally, a characteristic singlet signal observed at *δ* 8.98 ppm attributed to the azo-methine (–CH=N–) protons. The aromatic proton signals are observed at *δ* 6.90–8 ppm.^55^ The ^13^C NMR spectrum presented in Fig. 3 shows the aromatic carbon signals at *δ* 160.20–118.19 ppm. The signals observed at *δ* 163.88 and δ165.90 ppm correspond to the phenolic C–OH and CH=N carbons, respectively. These findings confirmed the proposed structure of the synthesized salphen-azobenzene ligand H_2_L_1_.

**Fig. 4:**
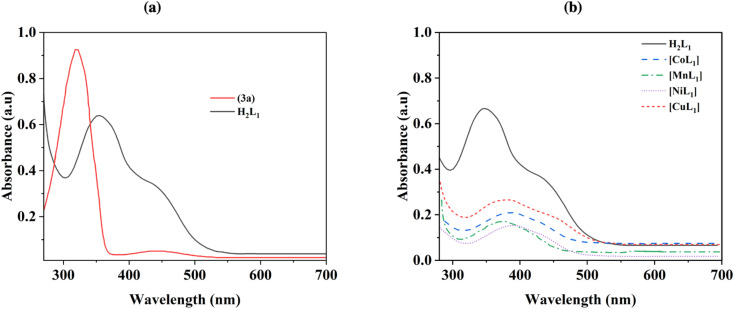
UV-Visible spectra of 3a and H_2_L_1_ (a), and its corresponding Co, Mn, Ni, and Cu complexes (b).

UV-Visible absorption spectra were collected for azobenzene (3a), salphen-azobenzene ligand H_2_L_1_, and its Co, Mn, Ni, and Cu complexes from 260–700 nm in chloroform solution at room temperature, and the obtained results are presented in Fig. 4. The azobenzene intermediate (3a) exhibited characteristic π–π* and n–π* electronic transitions, with the prominent *trans* π–π* absorption band appearing sharply at 350 nm (Fig. 4a). While the spectrum of ligand H_2_L_1_ displayed broader absorption bands that were red-shifted, which probably attributed the extension of the conjugated π system. The *trans* π–π* transition was observed at around 390 nm (Fig. 4a). Upon complexation of ligand H_2_L_1_ with Co(ii), Mn(ii), Ni(ii) and Cu(ii), the spectra of [CoL_1_], [MnL_1_], [NiL_1_] and [CuL_1_] showed a perturbation of the ligand-centered orbitals as evidenced by decreased intensities and shifting of the π–π* and n–π* bands to longer wavelengths. This suggests metal–ligand orbital mixing upon coordination. Additionally, d–d transition bands ascribed to the metal ions emerged in the higher wavelength region, observed at around 485 nm for [CoL_1_], 530 nm for [MnL_1_], 550 nm for [NiL_1_] and 525 nm for [CuL_1_] (Fig. 4b).

**Fig. 5:**
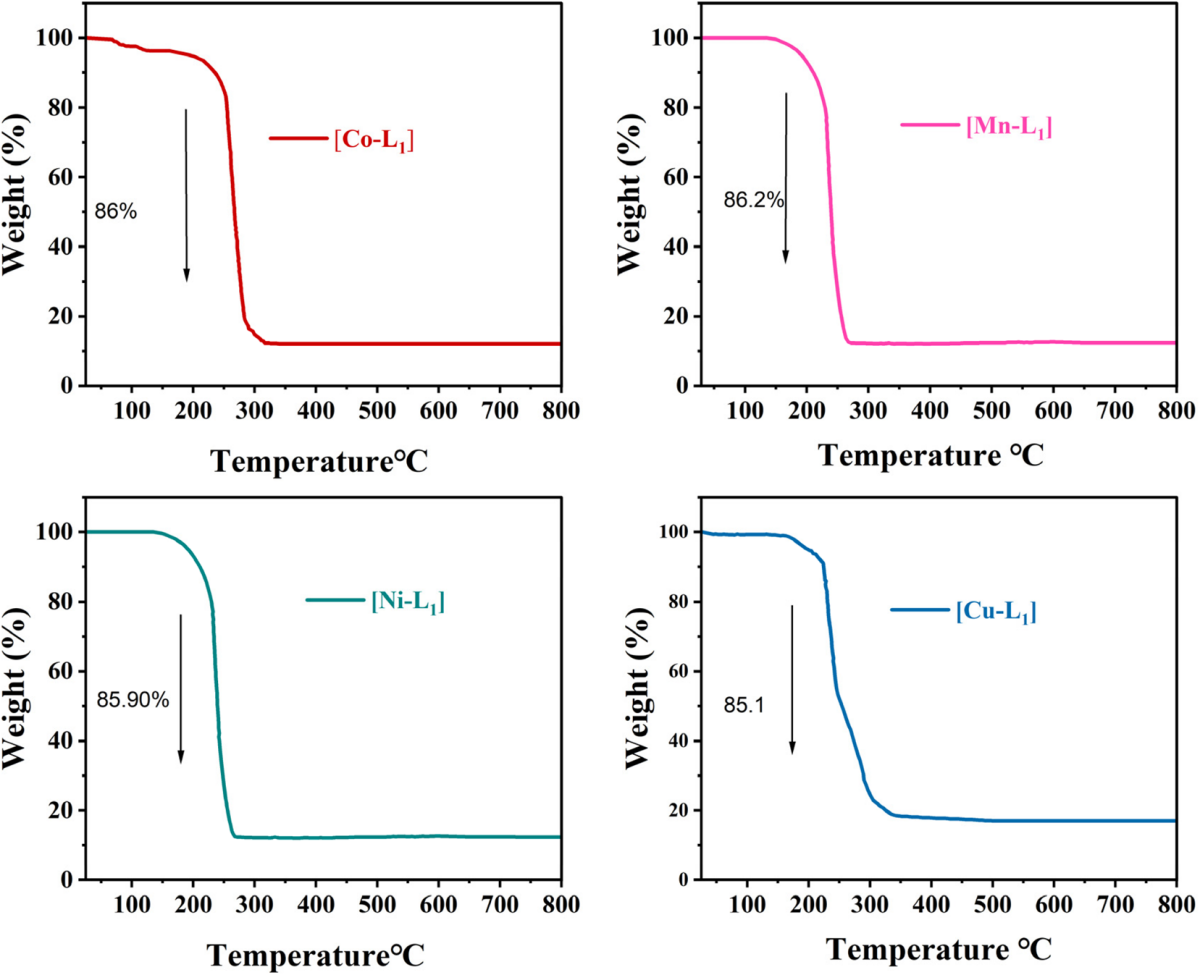
TGA curves of [CoL], [MnL], [NiL], and [CuL] complexes under air.

The thermal behavior of the complexes [CoL_1_], [MnL_1_], [NiL_1_], and [CuL_1_] was analyzed using thermogravimetric analysis (TGA) over a temperature range of 30–800 °C. The obtained results are displayed in Fig. 5. The initial weight loss step observed below 200 °C, which can be attributed to the evaporation of physically adsorbed water and solvent. The second weight loss step observed after 200 °C attributed to the breakdown of the organic ligand. Complexes [CoL_1_] and [NiL_1_] showed slight thermal stability compared to [CuL_1_] and [MnL_1_]. The complete breakdown of the salphen-azobenzene ligand occurred in the temperature ranges of 200–330 °C for [NiL] (85.90 wt%), 200–320 °C for [CoL_1_] (86 wt%), 200–270 °C for [MnL_1_] (86.20 wt%), and 200–260 °C for [CuL_1_] (85.1 wt%).

**Fig. 6:**
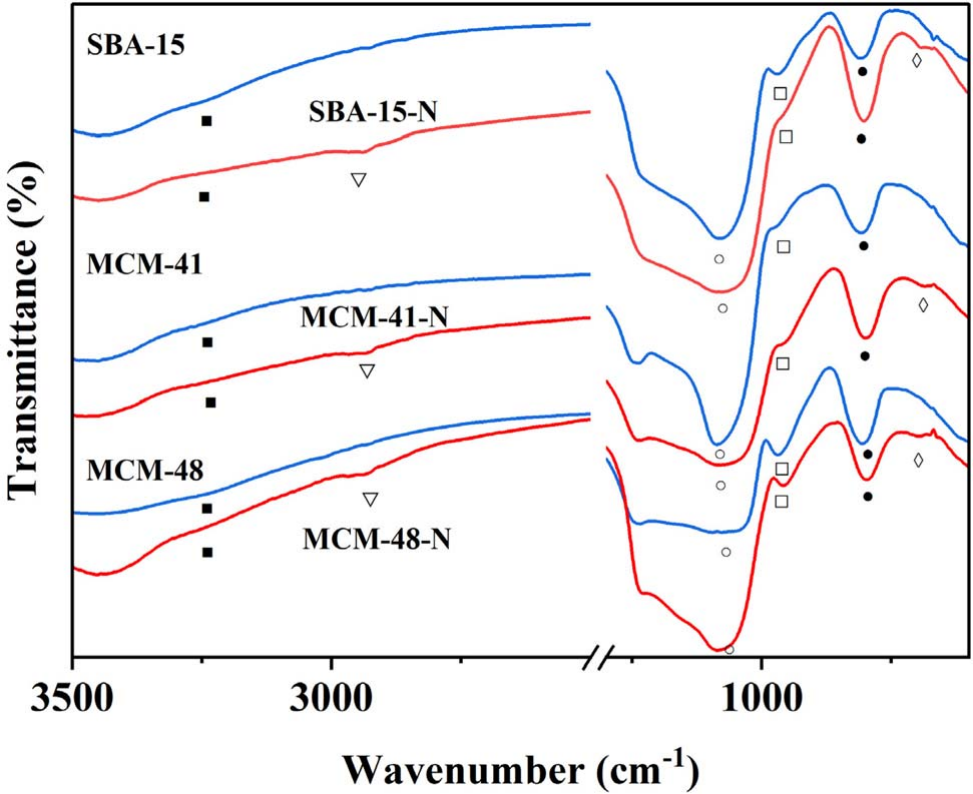
FTIR of SBA-15, MCM-41, and MCM-48 and modified silica SBA-15-N, MCM-41-N, and MCM-48-N.

Amino-functionalized materials were prepared by grafting aminopropyltriethoxysilane (APTES) onto the surface of SBA-15, MCM-41, and MCM-48, according to literature procedures.^52^ The FTIR spectra of the commercial silica materials (*i.e.*, SBA-15, MCM-41, and MCM-48), and their corresponding modified silica materials (*i.e.*, SBA-15-N, MCM-41-N, and MCM-48-N) are presented in Fig. 6. In the fingerprint region (700–1300 cm^−1^) of the mesoporous silica spectra, the symmetric (○) and asymmetric (●) stretching vibrations of the Si–O–Si linkages forming the silica frameworks showed two bands at ∼1045 cm^−1^ and 800 cm^−1^, respectively (Fig. 6). Upon grafting of APTES onto the silica surfaces, the intensities of both peaks corresponding to the OH stretching (■) and bending (□) vibrations noticeably decreased. This reduction occurred because the silica surface silanol groups (Si–OH) transformed to Si–O–Si–(CH_2_)_3_–NH_2_ after successful reaction with APTES. Additionally, a new peak at ∼2980 cm^−1^ (∇) appeared, which can be assigned to the CH_2_ groups of the grafted APTES. Further peaks (◊) at ∼700 cm^−1^ can be attributed to the stretching vibrations of the N–H bond of the APTES. All these findings demonstrate the successful covalent attachment of APTES onto the surfaces of the silica materials.

**Fig. 7:**
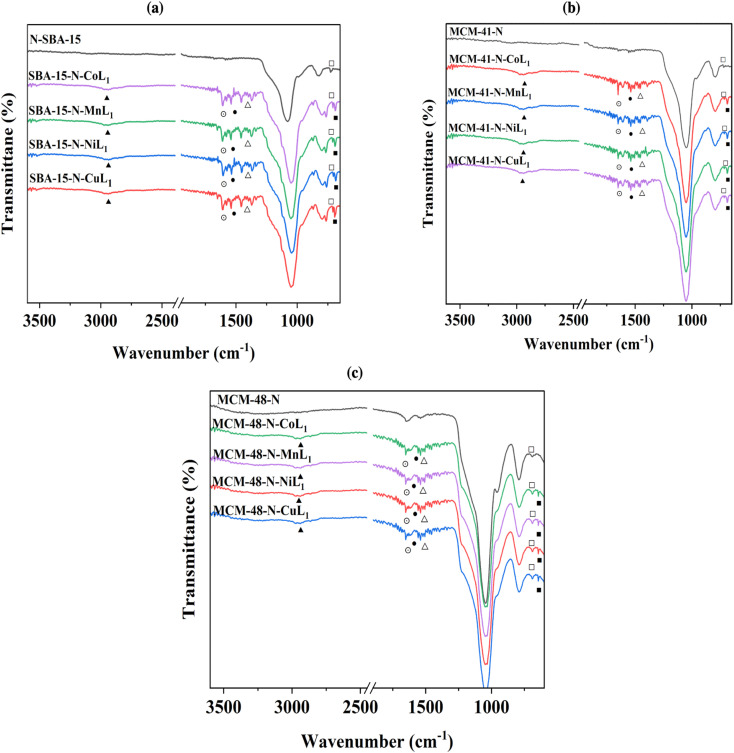
FTIR spectra of SBA-15-N-ML_1_ (a), MCM-41-N-ML_1_ (b), and MCM-48-N-ML_1_ (c), with M = Co, Mn, Cu or Ni.

After the addition of [ML_1_] complexes to the silica materials, FTIR analysis were performed to the obtained materials Silica-N-ML (*i.e.*, Silica: SBA-15, MCM-41, and MCM-48), and the obtained results are presented in Fig. 7a–c. The intensity of the peaks around 2980–3000 cm^−1^ (▲) corresponding to C–H increased compared to the same peaks in Silica-N. This can be due to the additional peaks of C–H groups in [ML_1_] complexes. Three peaks were observed at ∼1506 cm^−1^ (⨀), ∼1400 (●), and ∼1370 (Δ) which can be assigned to C=N, C=C, and N=N bonds in the metal complexes, respectively. The N–H bending (□) of the NH_2_ group was shifted to a lower frequency of ∼675 cm^−1^ after the addition of [ML_1_] complexes, which can be due to the formation of a coordination bond between the electron lone pair of NH_2_ groups and vacant orbital of the metal's ions (*i.e.*, Co, Mn, Ni, and Cu). Moreover, other tiny peaks appeared between 525 and 550 cm^−1^ (■), can be attributed to metal–O bonds. Co–O peak appeared at ∼542 cm^−1^, Mn–O at ∼547 cm^−1^, Ni–O at ∼556 cm^−1^, and Cu–O at ∼525 cm^−1^.

These observations confirmed the successful immobilization of [CoL_1_], [MnL_1_], [NiL_1_], and [CuL_1_] complexes onto the silica surfaces.

**Fig. 8:**
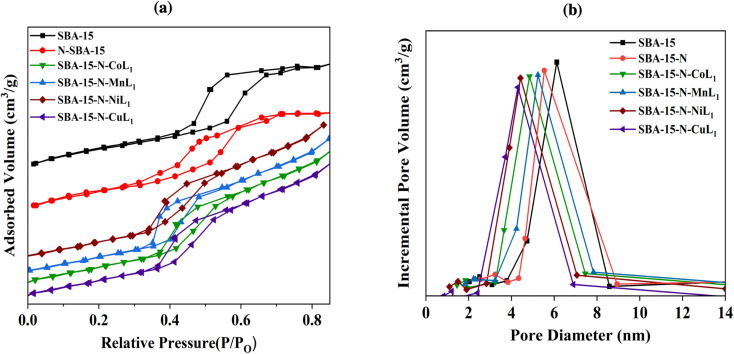
BET isotherms (a) and pore size distribution curves (b) of samples SBA-15, SBA-15-N, and SBA-15-N-ML_1_ (M: Mn, Co, Ni, or Cu).

**Fig. 9:**
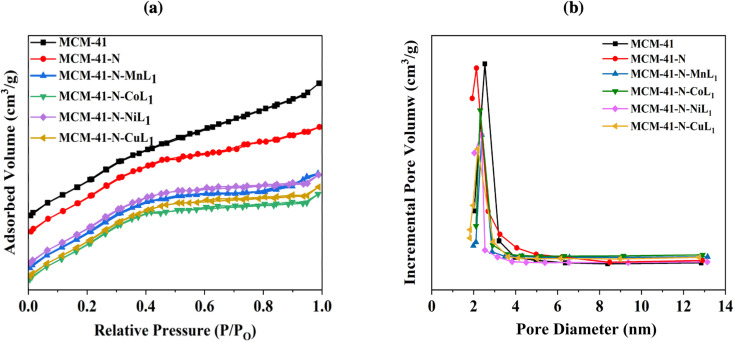
BET isotherms (a) and pore size distribution curves (b) of samples MCM-41, MCM-41-N, and MCM-41-N-ML_1_ (M: Mn, Co, Ni, or Cu).

**Fig. 10:**
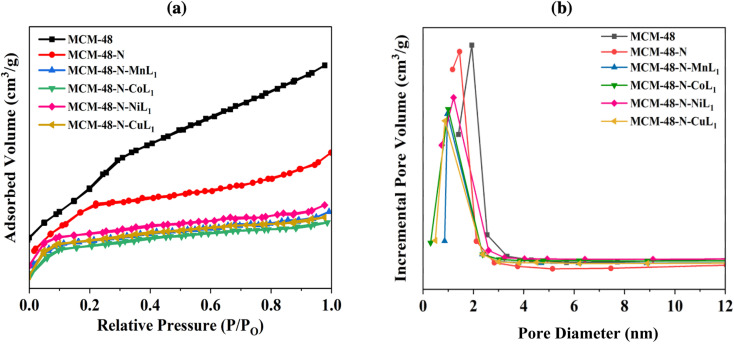
BET isotherms (a) and pore size distribution curves (b) of samples MCM-48, MCM-48-N, and MCM-48-N-ML_1_ (M: Mn, Co, Ni, or Cu).

N_2_ adsorption/desorption analysis was carried out to determine the surface area, pore size, and pore volumes of the different mesoporous silica materials before and after functionalization and immobilization of the metal complexes [ML_1_]. The average pore diameter and pore volume were derived using the Barrett, Joyner, and Halenda (BJH) method. Fig. 8, 9 and 10 present the resulting Brunauer, Emmett, and Teller (BET) isotherms and the pore size distribution curves of commercial silica (*i.e.*, SBA-15, MCM-41, MCM-48) as well as the amino-functionalized silica (*i.e.*, SBA-15-N, MCM-41-N, MCM-48-N), and the silica supported metallosalphen-azobenzene complexes Silica-N-ML_1_ (M: Co, Mn, Ni, or Cu; Silica: SBA-15, MCM-41, or MCM-48). Moreover, Table 1 summarizes the textural properties of all samples.

**Table 1:** Textural properties of commercial silica (SBA-15, MCM-41 & MCM-48), amino-functionalized silica (SBA-15-N, MCM-41-N & MCM-48-N), and silica supported metallosalphen-azobenzene complexes (SBA-15-N-ML_1_, MCM-41-N-ML_1_ & MCM-48-N-ML_1_, M: Mn, Co, Ni, or Cu)

Samples	*S*_BET_ a BJH^b^ (m^2^ g^−1^)	Pore diameter BJH^b^ (nm)	Pore volume BJH^b^ (cm^3^ g^−1^)
SBA-15	663.19	5.60	0.61
SBA-15-N	320.90	4.80	0.46
SBA-15-N-CoL_1_	248.30	3.88	0.37
SBA-15-N-Mn L_1_	249.20	3.98	0.38
SBA-15-N-NiL_1_	247.20	3.85	0.36
SBA-15-N-CuL_1_	247.60	3.88	0.37
MCM-41	1130.00	2.40	0.65
MCM-41-N	455.80	1.8	0.27
MCM-41-N-CoL_1_	372.90	1.50	0.19
MCM-41-N-MnL_1_	380.18	1.50	0.18
MCM-41-N-NiL	378.20	1.48	0.17
MCM-41-N-CuL_1_	377.99	1.47	0.17
MCM-48	1000.00	2.70	0.56
MCM-48-N	510.08	1.60	0.39
MCM-48-N-CoL_1_	406.05	0.98	0.21
MCM-48-N-MnL_1_	409.03	1.00	0.23
MCM-48-N-NiL_1_	406.00	0.97	0.21
MCM-48-N-CuL_1_	405.01	0.96	0.20

a Brunauer, Emmett, and Teller.

b Barrett, Joyner, and Halenda.

The obtained N_2_ adsorption/desorption isotherms of all samples demonstrated type IV isotherms with H^1^ hysteresis loops, indicating the maintenance of the mesoporous structures after the amino-functionalization and [ML] complexes immobilization (Fig. 8a). Commercial SBA-15 had a BET surface area of approximately 663.19 m^2^ g^−1^ and pore volume of 0.61 cm^3^ g^−1^ (Table 1). Its pore size distribution curve exhibited a peak at ∼5.6 nm (Fig. 8b). After APTES grafting, SBA-15-N's surface area and pore volumes decreased to 320.90 m^2^ g^−1^ and 0.46 cm^3^ g^−1^, respectively, with an average pore diameter of ∼4.8 nm (Fig. 8b), indicating a successful grafting of APTES onto SBA-15 surface.

After addition of [ML_1_], the surface area, pore size, and pore volume of SBA-15-N were all decreased (Table 1). Specifically, the surface areas of SBA-15-N-CoL_1_, SBA-15-N-MnL_1_, SBA-15-N-NiL_1_, and SBA-15-N-CuL_1_ were decreased to 248.30 m^2^ g^−1^, 249.20 m^2^ g^−1^, 247.20 m^2^ g^−1^ and 247.60 m^2^ g^−1^, respectively. Pore sizes of SBA-15-N-CoL_1_, SBA-15-N-MnL_1_, SBA-15-N-NiL_1_, and SBA-15-N-CuL_1_ were reduced to 3.88 nm, 3.98, 3.85 and 3.88 nm, respectively (Table 1). Additionally, pore volumes of SBA-15-N-CoL_1_, SBA-15-N-MnL_1_, SBA-15-N-NiL_1_, and SBA-15-N-CuL_1_ reduced to 0.37 cm^3^ g^−1^, 0.38 cm^3^ g^−1^, 0.36 cm^3^ g^−1^ and 0.37 cm^3^ g^−1^, respectively. This finding indicated a successful immobilization of the [ML_1_] complexes onto SBA-15-N surface.

Similar trends were observed for the MCM-41 samples. The commercial MCM-41 exhibited a BET surface area of approximately 1130.00 m^2^ g^−1^, with a pore volume of 0.65 cm^3^ g^−1^ (Table 1). The pore size distribution of MCM-41 showed a sharp peak centered around 2.40 nm (Fig. 9b), confirming a uniform pores size distribution. After the amino-functionalization of MCM-41 with APTES (N-MCM-41), the surface area was decreased to approximately 455.80 m^2^ g^−1^. The average pore diameter of N-MCM-41 was also decreased to 1.8 nm (Fig. 9b). Additionally, the average pore volume of MCM-41-N was reduced to 0.27 cm^3^ g^−1^. These findings indicated the successful grafting of APTES onto MCM-41 surface.

Furthermore, similar behavior was observed after the introduction of the metal complexes [ML_1_] into MCM-41-N. Specifically, the surface area of MCM-41-N was reduced to 380.18 m^2^ g^−1^, 372.90 m^2^ g^−1^, 378.20 m^2^ g^−1^, and 377.98 m^2^ g^−1^ for samples MCM-41-N-CoL_1_, MCM-41-N-MnL_1_, MCM-41-N-NiL_1_, and MCM-41-N-CuL_1_, respectively (Table 1). The pore sizes were also reduced to 1.50 1.50 nm, 1.48 nm, and 1.47 nm, respectively. Additionally, the pore volumes were reduced to 0.18 cm^3^ g^−1^, 0.19 cm^3^ g^−1^, 0.17 cm^3^ g^−1^, and 0.17 cm^3^ g^−1^, respectively (Fig. 9b).These results indicated the successful immobilization of the [ML_1_] complexes onto MCM-41-N surface.

Comparable results were found for the MCM-48 samples. The commercial MCM-48 exhibited a BET surface area of approximately 1000.00 m^2^ g^−1^, and an average pore volume of 0.56 cm^3^ g^−1^ (Table 1). The pore size distribution of MCM-48 showed a sharp peak at 2.7 nm (Fig. 10b), confirming uniform mesopores. After grafting APTES onto surface of MCM-48 its surface dramatically decreased to 510 m^2^ g^−1^. Similarly, the average pore diameter and average pore volume of N-MCM-48 was measured to be approximately 1.6 nm and 0.39 cm^3^ g^−1^, respectively (Fig. 10b, Table 1). This result indicated the successful grafting of APTES onto MCM-48 surface. As expected, after the incorporation of [ML_1_] complexes into MCM-48-N the surface area was decreased again to 409 m^2^ g^−1^, 406 m^2^ g^−1^, 406 m^2^ g^−1^ and 405 m^2^ g^−1^ for samples MCM-48-N-CoL_1_, MCM-48-N-MnL_1_, and MCM-48-N-NiL_1_, and MCM-48-N-CuL_1_, respectively (Table 1). In addition, the average pore diameter of the obtained samples were also reduced to 1 nm, 0.98 nm, 0.96 nm, and 0.97 nm respectively (Fig. 10b). Furthermore, similar behavior was observed for the average pore volumes of the prepared samples. The average pore volume of the final materials was also reduced to 0.23 cm^3^ g^−1^, 0.23 cm^3^ g^−1^, 0.20 cm^3^ g^−1^, and 0.21 cm^3^ g^−1^, respectively (Table 1). These findings indicate a successful immobilization of the metal complexes [ML_1_] onto the amino-functionalized MCM-48-N surface.

**Fig. 11:**
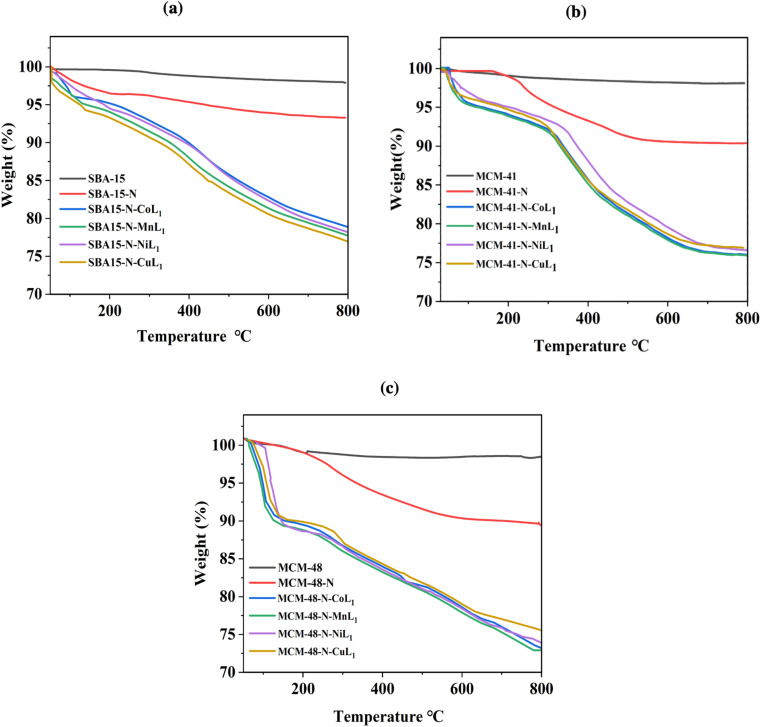
TGA thermographs of SBA-15 samples (a), MCM-41 samples (b), and MCM-48 samples (c).

TGA analysis was conducted on all silica samples, before and after the incorporation of the metallosalphen-azobenzene complexes to investigate their thermal behavior and determine the [ML_1_] content in silica. The obtained results are presented in Fig. 11a–c. The thermograms of all samples showed two main steps of weight loss. The initial step below 220 °C presents a weight loss of 2–5%, which is attributed to absorbed solvents and water residues. The second step observed above 220 °C related to the decomposition of the salphen-azobenzene ligand and APTES linker. Notably, the decomposition step of all silica supported [ML_1_] complexes was started at around 200 °C but extended to around 750 °C, compared to unsupported [ML_1_] which exhibited a rapid decomposition between 200 °C and 300 °C. This suggests that the silica wall acted as a thermal insulator for the [ML_1_] complexes.

The thermogravimetric analysis of SBA-15-N, MCM-41-N and MCM-48-N was performed to serve references. For all three materials, the APTES-propylamine groups decomposed between 330–450 °C, with a weight loss of around 6–7%. Specifically, SBA-15-N exhibited a weight loss of 4.5% in this temperature range. MCM-41-N showed a similar weight loss of 4.3%. Similar weight loss of 4.7% was also observed for MCM-48-N. The remaining insignificant weight loss occurring up to 850 °C for all materials could be assigned to condensation reactions between silanol groups on the silica surface.^56,57^

The weight loss observed between 220–750 °C corresponds to the decomposition of the salphen-azobenzene ligand and APTES linker, with organic weight losses (APTES + L) of 13.10% for SBA-15-N-CoL, 13.6% for SBA-15-N-MnL, 12.75% for MCM-41-N-CoL_1_, 13.5% for MCM-41-N-MnL_1_, 12.94% for MCM-48-N-CoL_1_ and 12.01% for MCM-48-N-MnL_1_. This is consistent with the ML_1_ content in silica determined to be in the range of 0.97–1.25 wt% as presented in Table 2, which is determined by ICP-MASS.

**Table 2:** Metal content determined by ICP-MASS

Samples	ML_1_ content in silica^a^ (wt%)
SBA-15-N-CoL_1_	1.25
SBA-15-N-MnL_1_	1.22
MCM-41-N-CoL_1_	1.23
MCM-41-N-MnL_1_	1.21
MCM-48-N-CoL_1_	1.22
MCM-48-N-MnL_1_	0.97

a Metal content determined by ICP-MASS.

**Fig. 12:**
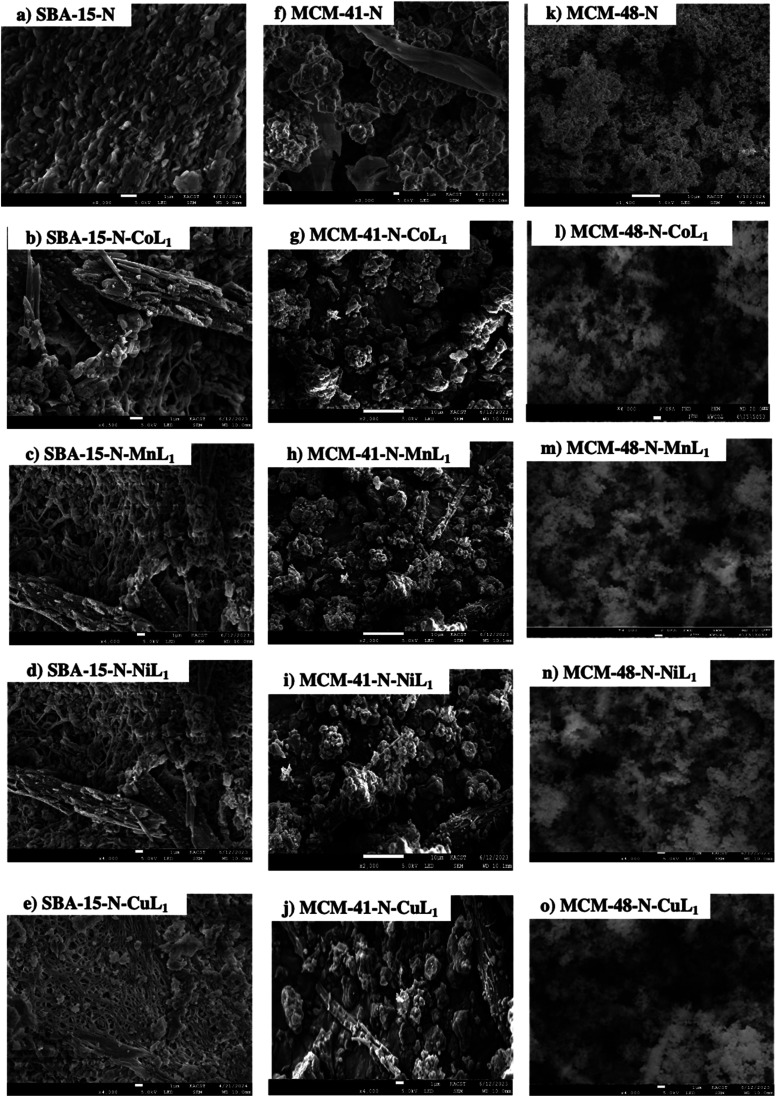
SEM micrographs of SBA-15-N (a), SBA-15-N-CoL_1_ (b), SBA-15-N-MnL_1_ (c), SBA-15-N-NiL_1_ (d), SBA-15-N-CuL_1_ (e), MCM-41-N (f), MCM-41-N-CoL_1_ (g), MCM-41-N-MnL_1_ (h), MCM-41-N-NiL_1_ (i), MCM-41-N-CuL_1_ (j), MCM-48-N (k), MCM-48-N-CoL_1_ (l), MCM-48-N-MnL_1_ (m), MCM-48-N-NiL_1_ (n), and MCM-48-N-CuL_1_ (o).

The morphology of the amino-functionalized silica SBA-15-N, MCM-41-N, and MCM-48-N, along with their corresponding Silica-N-ML samples were investigated using scanning electron microscopy (SEM). The obtained results are presented in Fig. 12. The SEM images SBA-15-N, MCM-41-N, and MCM-48-N (a, f and k), depict particles with length ranging 0.5–5.0 μm, 0.10–0.27 μm, and 0.12–0.24 μm. Their corresponding SBA-15-N-ML_1_ (b–e), MCM-41-N-ML_1_ (g–j), and MCM-48-N-ML_1_ (l–o) depict particles with length ranging (0.5–0.22), (0.29–0.21), and (0.19–0.22) μm respectively. Samples SBA-15-N, MCM-41-N, and MCM-48-N exhibited some degree of aggregation and formation of tubular agglomerates due to formation of hydrogen bonding between the amine groups and the surface silanols. However, after the addition [ML_1_] complexes (Fig. 12b–e, g–j and l–o) the aggregation was reduced, and isolated and smaller particles were observed. This decrease provides direct evidence that coordination bonding stabilizes the nanoparticles. Without crosslinking, Silica-N particles could aggregate and grow larger over time. However, the metal–amine coordination bonds restrict this by rigidly connecting particles at a smaller set size. Therefore, the size reductions upon addition of the ML_1_ complexes directly support our hypothesis that coordination bonding counteracts aggregative processes and enhances nanoparticle stability.

**Fig. 13:**
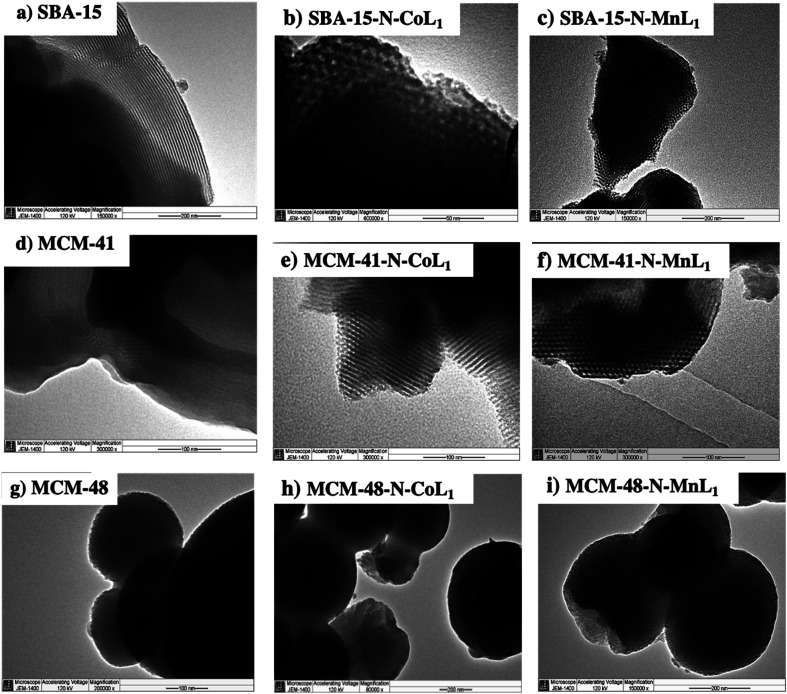
TEM images of commercial silica SBA-15 (a), MCM-41 (d), and MCM-48 (g), and silica supported [CoL] (b, e and h) and [MnL] (c, f and i).

Transmission electron microscopy (TEM) was used to visualize the nanostructures and dispersion of [ML_1_] complexes trough silica surface. However, TEM images were obtained only for Silica-N-CoL_1_ and Silica-N-MnL_1_ and their corresponding silica materials. Because these two catalysts exhibited higher catalytic activity in the oxidation reaction of cyclohexane compared to their Ni and Cu analogues. The obtained TEM images are presented in Fig. 13. Images (a) and (d) display the parallel mesoporous channels, characteristic of the highly nano-ordered SBA-15 and MCM-41 materials, respectively. The TEM images obtained for these two materials after the incorporation of [CoL_1_] (Fig. 13b and e) and [MnL_1_] (Fig. 13c and f) revealed the preservation of the silica nanostructure. More importantly, the absence of any dark spots in the TEM images of Silica-N-CoL_1_ and Silica-N-MnL_1_ (Silica: SBA-15 or MCM-41) indicates the high dispersion of [CoL_1_] and [MnL_1_] through SBA-15 and MCM-41 surface.

The obtained TEM images showed a spherical morphology of MCM-48 particles (Fig. 13g), with the average particle's diameters of 245 nm. However, images at more than 200 000× were not taken to visualize clearly the very tiny nanochannels of MCM-41 material.^58^ After the addition of [CoL_1_] and [MnL_1_] to prepared MCM-4-N-CoL_1_ and MCM-4-N-MnL_1_, TEM images (Fig. 13h and i) revealed the preservation of the spherical morphology of MCM-48 with average particles diameter of 220 nm and 222 nm, respectively. Moreover, the absence of any dark spots in the obtained TEM images indicates high dispersion of [CoL_1_] and [MnL_1_] through MCM-48 surface.

**Fig. 14:**
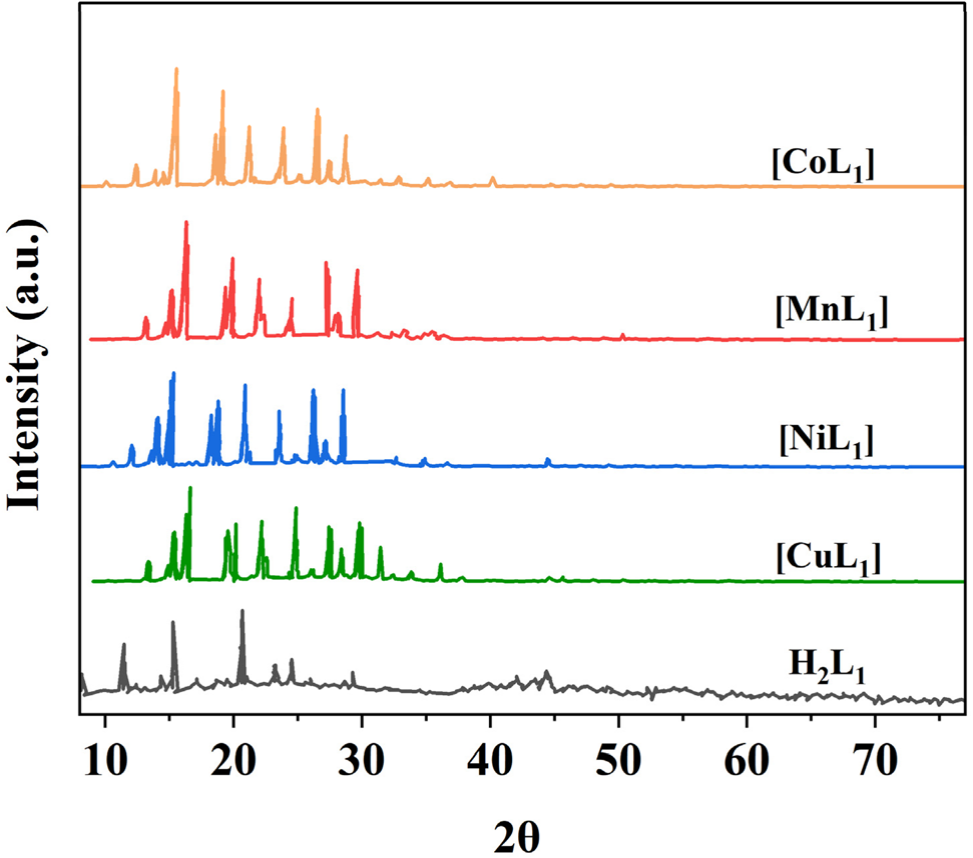
XRD patterns of salphen-azobenzene ligand H_2_L_1_ and its metal complexes [ML_1_] (M: Mn, Co, Ni, and Cu).

Powder XRD was performed for the salphen-azobenzene ligand H_2_L_1_ and its metal complexes [ML_1_] where M = Mn, Co, Ni, Cu (Fig. 14). All samples presented distinct peaks corresponding to their crystalline nature. This confirms the formation of metal complexes [ML_1_]. The complexes display less intense peak reflections compared to the free ligand which indicates that the crystallinity of the H_2_L_1_ decreases upon complexation with M(ii) ion = Mn, Co, Ni, and Cu. This observation agrees with previous reports on the impact of metal coordination on ligand crystallinity.^59,60^

**Fig. 15:**
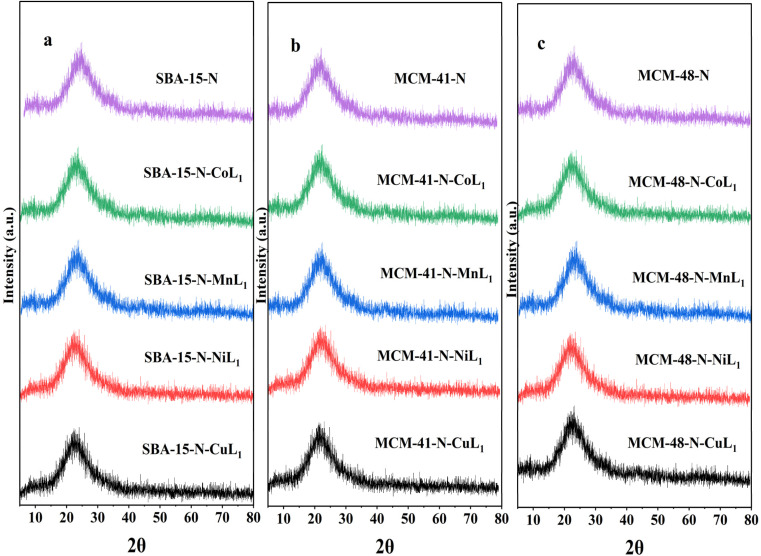
XRD patterns of SBA-15-N and SBA-15-N-ML1 (a), MCM-41-N and MCM-41-N-ML1 (b), MCM-48-N and MCM-48-N-ML1 (c), with M = Mn, Co, Ni, or Cu.

After incorporation of these metal complexes [ML_1_] into silica Silica-N-ML_1_ (Silica: SBA-15, MCM-41 or MCM-48; M: Mn, Co, Ni or Cu), as shown in Fig. 15, all samples displayed a single broad peak around 2*θ* = 22.9°, characteristic of amorphous silica. Notably, no distinctive peaks for crystalline complexes [ML_1_] were observed. This indicates the metal complexes were highly dispersed on the mesoporous silica surfaces.^61^ This is in agreement with the TEM results described above. This confirms our approach to prepare Silica-N-ML_1_ materials with only isolated [CoL_1_], [MnL_1_], [NiL_1_] and [CuL_1_] molecules coordinated to surface NH_2_ groups, without aggregation phase, and to remove the free molecules by filtration and frequent washing process.

### 3.2 Oxidation of cyclohexane

The catalytic activity of the prepared Silica-N-ML_1_ materials was evaluated in the oxidation reaction of cyclohexane to produce KA oil. The reaction was performed in a sealed autoclave. In this study different parameters such as type of oxidant, temperature, reaction time, catalyst dose, solvent. The reaction was monitored by gas chromatography (GC) using chlorobenzene as an internal standard.

#### 3.2.1 Effect of oxidant

Different oxidants were evaluated in this study including hydrogen peroxide (H_2_O_2_), *tert*-butyl hydroperoxide (TBHP), and *meta*-chloroperoxybenzoic acid (*m*-CPBA). Based on previous results obtained by our research group and reported in the literature.^62,63^ The reactivity of these oxidant can be classified in the following order *m*-CPBA > TBHP > H_2_O_2_ > O_2_. The reaction was carried out following a procedure reported by Arumugam *et al.*^53^ by adding 1.0 mL of cyclohexane (10 mmol), 100 mg of the catalyst, 1.0 mL of chlorobenzene (10 mmol) as an internal standard, 5.0 mL of acetonitrile as solvent, and the oxidant to a 50 mL sealed autoclave. After heating the autoclave at 60 °C for 6 h, 1.0 mL aliquots were withdrawn from the reaction mixture and filtered using a hydrophobic membrane to remove solids. The 10 μL of filtered aliquots were analyzed by GC without dilution. This allowed the determination of cyclohexane conversion and KA Oil selectivity.

##### 3.2.1.1 *m*-CPBA

Table 3 summarize the obtained results with different Silica-N-ML_1_ catalysts compared to unsupported [ML_1_], using 1.5 eq. of *m*-CPBA (2.5 g, 15 mmol) as an oxidant. Without a catalyst (entry 1), no conversion was observed. The unsupported catalysts [CoL_1_] (entry 2), [MnL_1_] (entry 3), [NiL_1_] (entry 4), and [CuL_1_] (entry 5) showed a cyclohexane conversion ranging from 40% to 55%, and KA oil selectivity in the range of 41–54%. The heterogeneous catalysts SBA-15-N-CoL_1_ (entry 6), SBA-15-N-MnL_1_ (entry 7), SBA-15-N-NiL_1_ (entry 8), and SBA-15-N-CuL_1_ (entry 9), exhibited higher conversion of 69–79%, and improved selectivity of 68–78%. When SAB-15 was replaced by MCM-41 the conversion and selectivity were slightly improved. 89%/90% and 85%/87% (conversion/selectivity) were obtained with MCM-41-N-CoL_1_ (entry 10) and MCM-41-N-MnL_1_ (entry 11) respectively. However, no significant improvement was observed with MCM-41-N-NiL_1_ (entry 12) and MCM-41-N-CuL_1_ (entry 13). Furthermore, when SBA-15 was replaced by MCM-48 the conversion and selectivity were slightly decreased. We can conclude that the best results were obtained with [CoL_1_] and [MnL_1_], using SBA-15 as a support. Compared to the literature (entries 18–21), despite performing the reaction at higher temperature (70–80 °C) and for more time (12–24 h) the obtained results are less important than those obtained in this work.

**Table 3:** Oxidation of cyclohexane over different catalysts using 1.5 eq. (2.50 g, 15 mmol) *m*-CPBA as an oxidant. Cyclohexane: 1.0 mL, catalyst: 100 mg, chlorobenzene: 1.0 mL, *T*: 60 °C, time: 6 h, solvent: 5 mL of acetonitrile

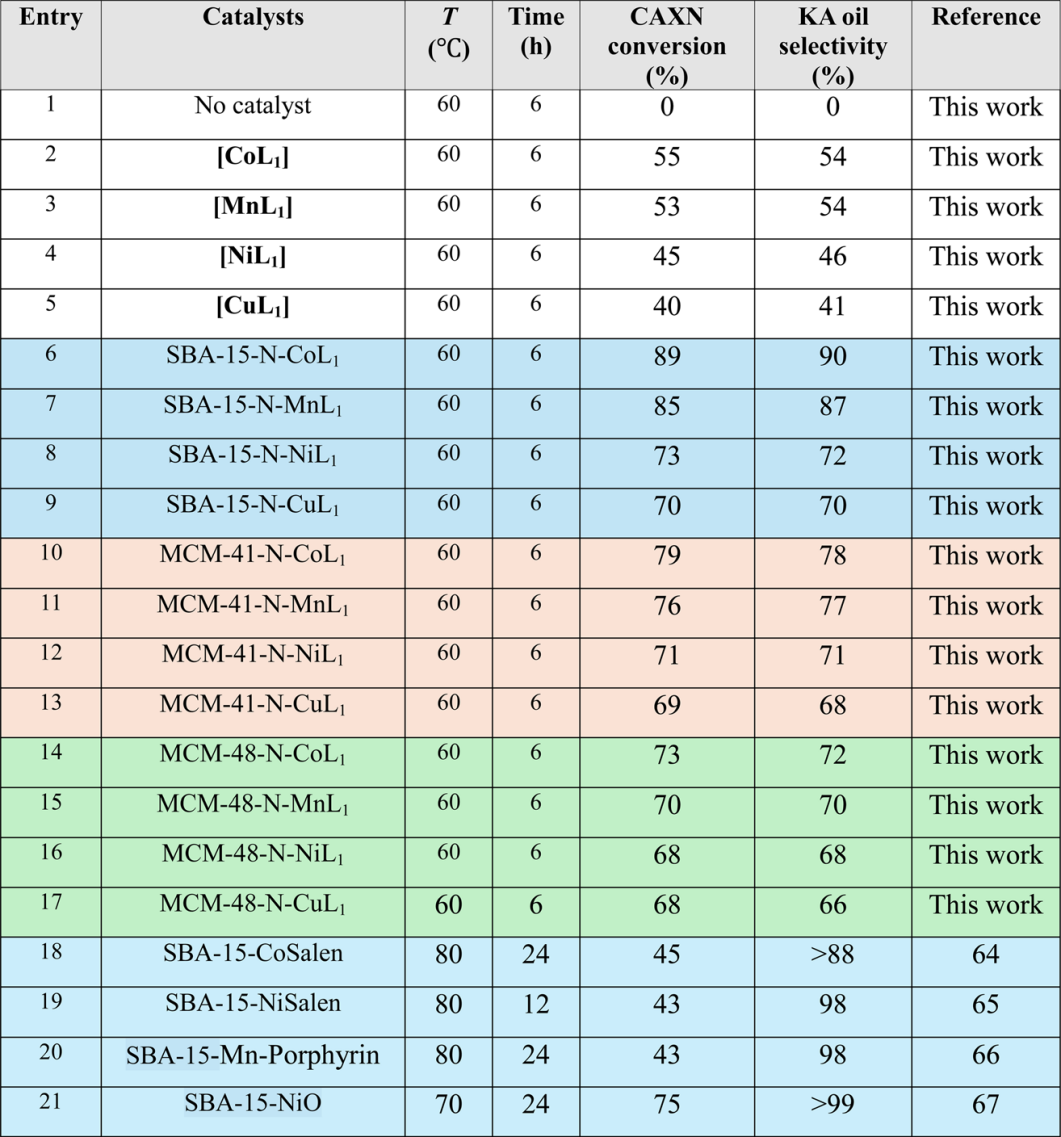

##### 3.2.1.2 TBHP

Table 4 summarize the obtained results with different Silica-N-ML_1_ catalysts compared to unsupported [ML_1_], using 2 eq. of TBHP (1.80 mL, 20 mmol) as oxidant. As expected, the conversion and selectivity results obtained with TBHP are generally lower than those obtained with *m*-CPBA. The unsupported complexes [ML_1_] (entries 2–5) showed low conversions ranging from 42 to 33%, and selectivity toward KA oil in the range of 40–32%. The silica supported complexes exhibited higher conversion and selectivity (entries 6–17), with cyclohexane conversion in the range of 60–70%, and KA oil selectivity between 70–62%. The best results were obtained with [CoL_1_] and [MnL_1_] supported on SBA-15 and MCM-41 (entries 6, 7, 10, and 11). Lower conversions and selectivity were obtained with the other catalytic systems. Literature catalysts (entries 18–21) such as SiO_2_-CoL and SiO_2_-Mn-salophen tested at different conditions showed conversions ranging from 40–31% and selectivity over 88–29%. Moreover, compared to the literature, low conversion was obtained even at higher temperatures 70–80 °C during 6 h (entries 18–20). Very low conversion and selectivity obtained at room temperature for 12 h using SiO_2_-Mn-salophen (entry 21).

**Table 4:** Oxidation of cyclohexane over different catalysts using 1.80 mL (20 mmol) of TBHP as an oxidant. Cyclohexane: 1.0 mL, catalyst: 100 mg, chlorobenzene: 1.0 mL, *T*: 60 °C, time: 6 h, solvent: 5 mL of acetonitrile

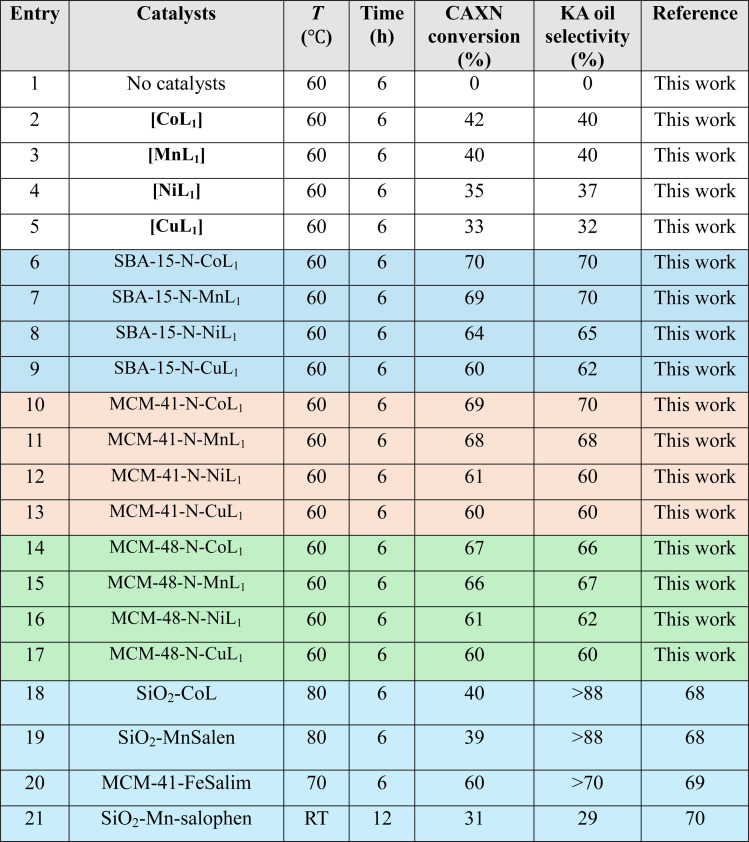

##### 3.2.1.3 H_2_O_2_

H_2_O_2_ was tested as an eco-friendly oxidant, using 2.0 mL (20 mmol) of 30% H_2_O_2_. However, SBA-15-CoL was chosen among the heterogeneous catalysts exhibited the best catalytic activity with *m*-CPBA and TBHP. The obtained results were compared with some selected results from the literature and presented in Table 5. 21% cyclohexane conversion and 50% KA oil selectivity were obtained with SBA-15-N-CoL_1_ as catalyst (entry 1). This result was similar to that obtained with Co-(complex) SiO_2_ at 70 °C (entry 2). Better results were obtained by increasing the time or/and temperature (entries 3 and 4). However, even using a cobalt-based catalyst, and increasing the reaction temperature to 100 °C, only 12% of cyclohexane was converted to product, with 80% selectivity (entry 5).

**Table 5:** Oxidation of cyclohexane over different catalysts using 2.0 mL (20 mmol) of 30% H_2_O_2_ as an oxidant. Cyclohexane: 1.0 mL, catalyst: 100 mg, chlorobenzene: 1.0 mL, *T*: 60 °C, time: 6 h, solvent: 5 mL of acetonitrile

Entry	Catalysts	*T* (°C)	Time (h)	CAXN conversion (%)	KA selectivity (%)	Reference
1	SBA-15-N-CoL_1_	60	6	21	50	This work
2	SiO_2_-CoComplex	70	6	20	55	71
3	MCM-41-CoSalen	60	12	45	50	72
4	MCM-41-MnSalen	80	12	37	70	73
5	SiO_2_-CoSalen	100	6	12	80	74

To better understand factors influencing the catalytic performance of SBA-15-N-CoL_1_, and to optimize the reaction conditions, other parameters were also investigated, such as the reaction temperature, reaction time, catalysts dose, amount of *m*-CPBA, and *cis*/*trans* isomerization of the azobenzene moiety of the ligand H_2_L_1_.

#### 3.2.2 Effect of temperature

**Fig. 16:**
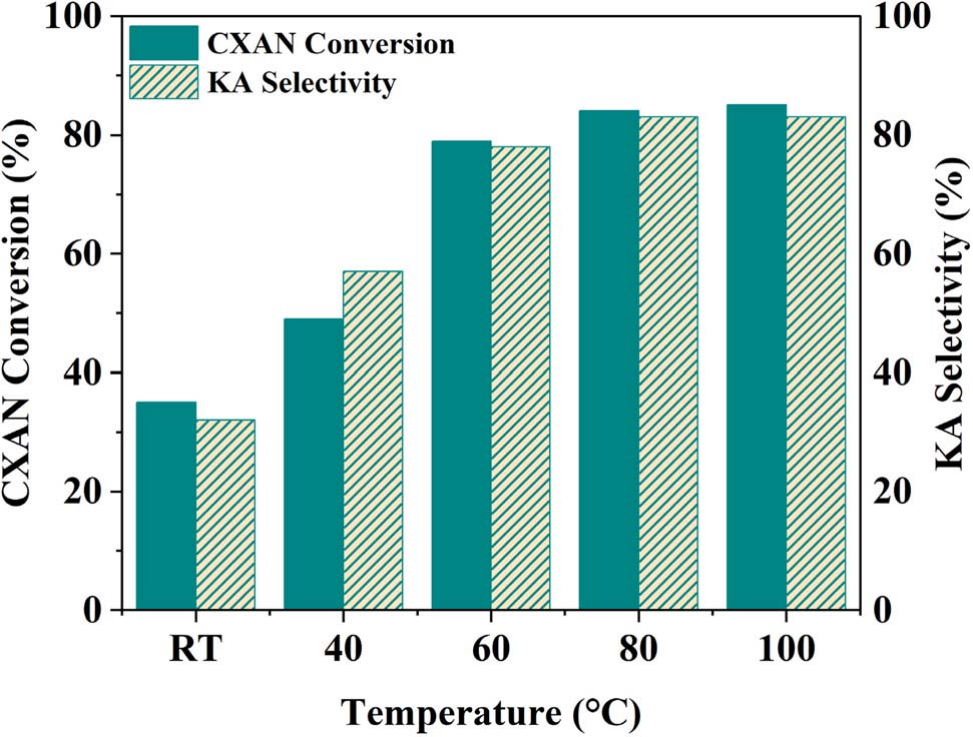
Effect of temperature on the cyclohexane oxidation over SBA-15-N-CoL_1_. Cyclohexane: 1.0 mL, catalyst: 100 mg, oxidant: 2.50 g of *m*-CPBA, chlorobenzene: 1.0 mL, time: 6 h, solvent: 5 mL of acetonitrile.

The effect of temperature on the oxidation reaction of cyclohexane to produce KA oil over SBA-15-CoL_1_ was investigated using *m*-CPBA as oxidant for 6 h, using *m*-CPBA (1.5 eq., 2.50 g, 15 mmol) as oxidant, with 1.0 mL of cyclohexane (10 mmol), 1.0 mL of chlorobenzene (10 mmol), 100 mg of SBA-15-N-CoL_1_, in 5 mL acetonitrile as a solvent at temperature from RT to 100 °C. The obtained results revealed that cyclohexane conversion was improved when the temperature was increased from room temperature (RT) to 100 °C (Fig. 16). At RT 35% of cyclohexane was converted to products with 32% selectivity toward KA oil. The conversion was gradually increased from 35% to 84% as the temperature increased from RT to 80 °C. However, raised temperature from 80 to 100 °C did not show any significant change in conversion only a small increment from 84% to 86%, while the selectivity remained at 83% conversion. This in agreement with previous results reported in the literature.^75,76^

#### 3.2.3 Effect of reaction time

**Fig. 17:**
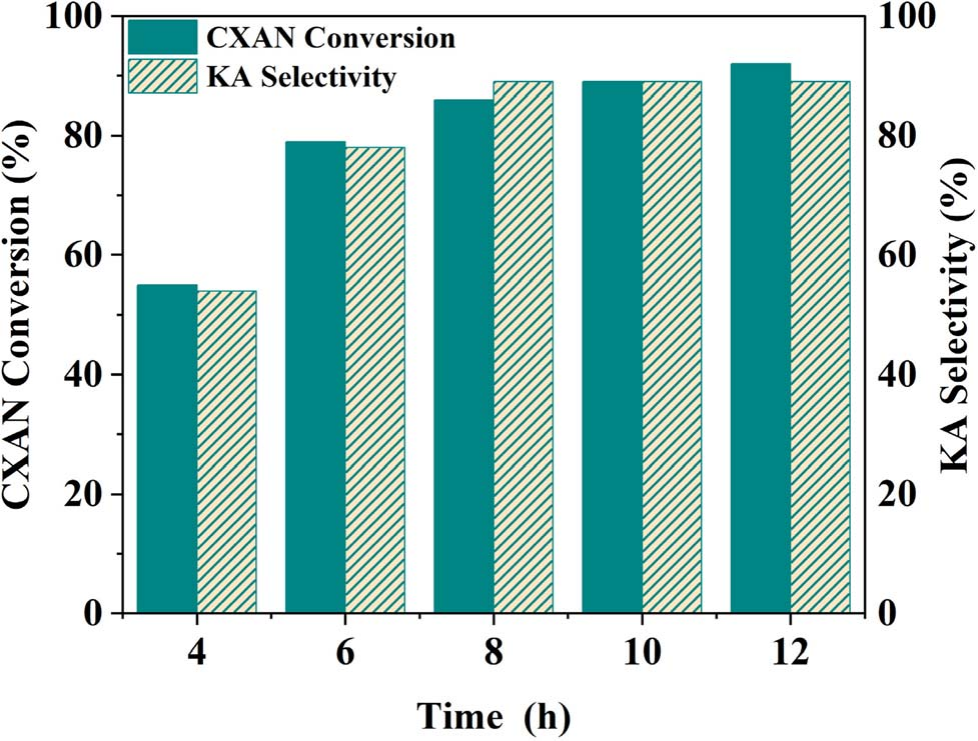
Effect of reaction time on the oxidation of cyclohexane. Cyclohexane: 1.0 mL, chlorobenzene: 1.0 mL, catalyst: 100 mg of SBA-15-N-CoL, oxidant: 2.50 g of *m*-CPBA, solvent: 5 mL of acetonitrile, *T*: 60 °C.

To optimize the reaction time, the cyclohexane oxidation reaction over SBA-15-CoL_1_ was performed in different reaction time, from 0 h to 12 h, using *m*-CPBA (1.5 eq., 2.50 g mg, 15 mmol) as oxidant, with 1.0 mL of cyclohexane (10 mmol), 1.0 mL of chlorobenzene (10 mmol), 100 mg of SBA-15-N-CoL_1_, in 5 mL acetonitrile as a solvent at 60 °C. As expected, the obtained results revealed that the cyclohexane conversion was gradually increased from 55% to 92% as seen in Fig. 17. However, the selectivity of KA oil initially increased and then remained constant at 89% with longer reaction times. This in agreement with previous results reported in the literature.^77^

#### 3.2.4 Effect of catalysts dose

**Fig. 18:**
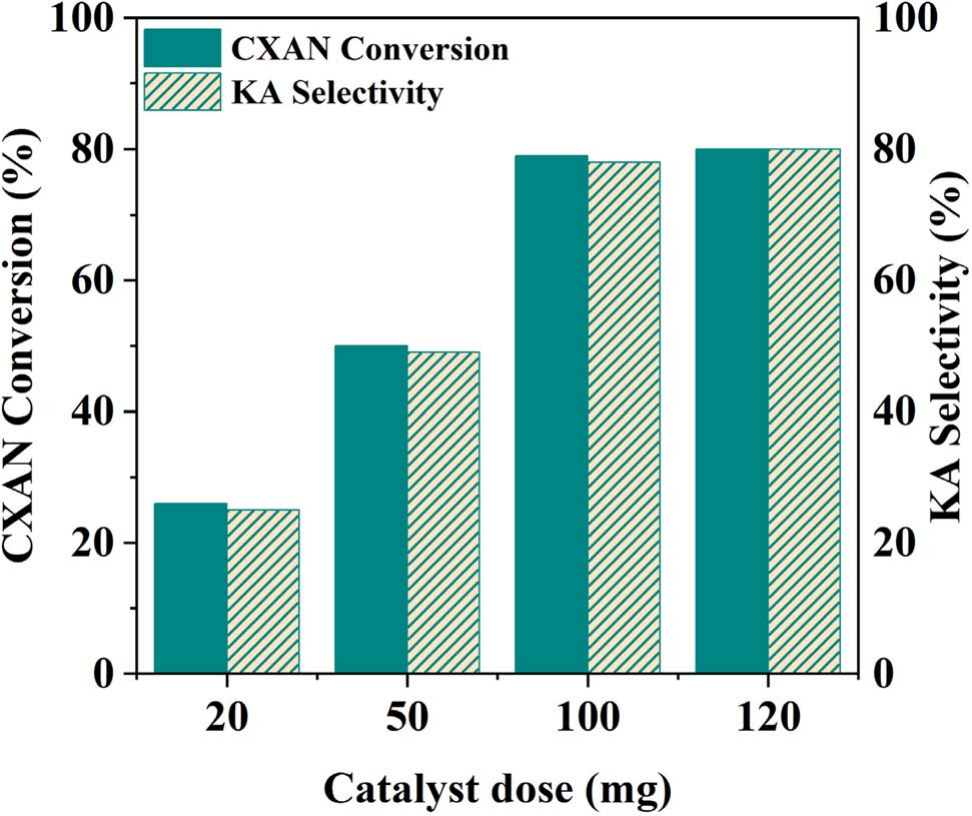
Effect of catalysts dose on the oxidation of cyclohexane. Cyclohexane: 1.0 mL, chlorobenzene: 1.0 mL, catalyst: 20–120 mg of SBA-15-N-CoL_1_, oxidant: 2.50 g of *m*-CPBA, solvent: 5 mL of acetonitrile, *T*: 60 °C, time: 6 h.

The effect of catalyst dose was studied over the range of 20–120 mg, using SBA-15-N-CoL_1_ as catalyst, *m*-CPBA (1.5 eq., 2.50 g, 15 mmol) as oxidant, with 1.0 mL of cyclohexane (10 mmol), 1.0 mL of chlorobenzene (10 mmol), in 5 mL acetonitrile as a solvent at 60 °C for 6 h. The obtained results are presented in Fig. 18. The obtained results showed that the cyclohexane conversion and KA oil selectivity were gradually increased from 25% and 25% to 79% and 78% as the catalyst dose increased from 20 to 100 mg, respectively. However, no significant change was observed in the conversion and selectivity when the catalyst amount increased from 100 mg to 120 mg. Therefore, a catalyst dose of 100 mg was determined to be optimal.

#### 3.2.5 Effect of amount of oxidant

**Fig. 19:**
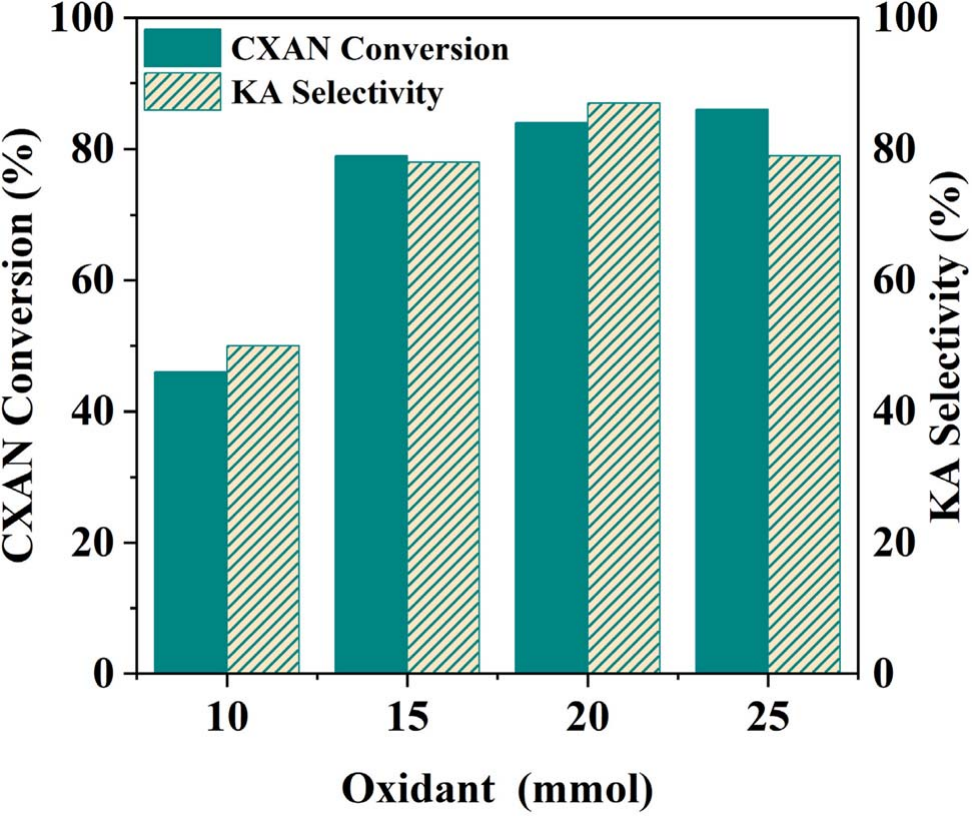
Effect of oxidant amount on the oxidation of cyclohexane. Cyclohexane: 1.0 mL, chlorobenzene: 1.0 mL, catalyst: 100 mg of SBA-15-N-CoL_1_, oxidant: 1–2.5 eq. of *m*-CPBA, solvent: 5 mL of acetonitrile, *T*: 60 °C, time: 6 h.

In this study, different amounts of *m*-CPBA (1–2.5 eq.) were used for the oxidation of cyclohexane to produce KA oil over SBA-15-N-CoL to determine the optimized dose. Using 100 mg of catalyst, with 1.0 mL of cyclohexane (10 mmol), 1.0 mL of chlorobenzene (10 mmol), in 5 mL acetonitrile as a solvent at 60 °C for 6 h. The obtained results are seen in Fig. 19. When the amount of *m*-CPBA was decreased to 1 eq. (10 mmol), both cyclohexane conversion and KA oil selectivity were decreased to 46% and 50%, respectively. Excessively increasing *m*-CPBA to 2.5 eq. (25 mmol) led to a slight increase in the conversion (86%) and selectivity (79%), compared to result obtained with 1.5 eq. of *m*-CPBA.

Furthermore, by-products of oxidation of cyclohexane include over-oxidized products resulting from oxidation of the cyclohexanone or cyclohexanol. Common by-products include cyclohexanone oxime, cyclohexenone, and dicarboxylic acids such as glutaric and adipic acid.^78,79^*m*-CPBA is commonly used as the oxidant for cyclohexane oxidation due to its ability to perform the reaction cleanly with few by-products such as dicarboxylic acid.^3,5^*m*-CPBA is commonly used as the oxidant for cyclohexane oxidation due to its ability to perform the reaction cleanly with few by-products such as dicarboxylic acid.^3,5^*m*-CPBA oxidizes cyclohexane to cyclohexanone and cyclohexanol in a stereospecific catalyst without cleaving the ring. This allows the reaction to obtain high yields of the desired mono-oxidation products with minimal over-oxidation.^80,81^ The ratio of cyclohexanone to cyclohexanol products (K/A ratio) is affected by several reaction conditions. Previous work has shown that increasing the amount of catalyst, leads to higher conversions but also increases over-oxidation reactions, lowering the K/A ratio.^82^ Extending the reaction time beyond 7 hours has a similar effect, as longer reactions enhance further oxidation of cyclohexanone and cyclohexanol into by-products.^81,83,84^ Other studies have also demonstrated the influence of reaction parameters on cyclohexane oxidation for example, Lesbani and coworker, found that temperatures below 80 °C using *m*-CPBA resulted in higher cyclohexanone selectivity while also suppressing by-product formation.^76^ Additionally, Maciuk *et al.* 2023, showed that the use of an *m*-CPBA to cyclohexane ratio of 1.5 : 1 improved conversion without significantly impacting selectivity or yield of by-products.^80^ Optimal conditions such as a catalyst dose of 100 mg, reaction time between 6–8 hours, and temperature below 80 °C, and a 1.5 : 1 *m*-CPBA : cyclohexane ratio can minimize by-product formation.

#### 3.2.6 Effect of *cis*/*trans* conformation of the catalyst

**Fig. 20:**
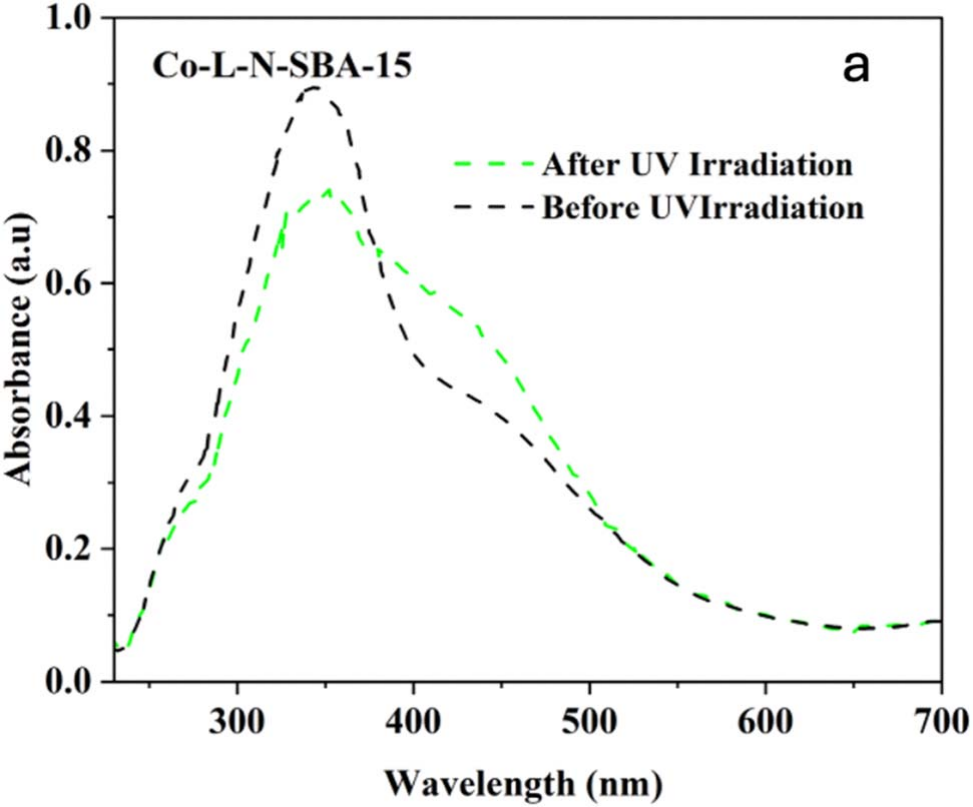
UV-Visible diffuse reflectance (DR) spectra of SBA-15-N-CoL_1_ before UV irradiation (black line) and after UV irradiation at 365 nm for 45 min (green line).

The *cis*/*trans* isomerization of the azobenzene in the catalyst SBA-15-N-CoL_1_ was confirmed by UV-Visible diffuse reflectance (DR) spectroscopy, and the obtained result presented in Fig. 20. Before UV irradiation, the DR spectrum showed the main characteristic absorption band of the *trans* configuration at 340 nm, which attributed to π–π* transition, with a small band of the *cis* configuration at 442 nm, which related to n–π* transition (black line). Upon exposure to UV light 365 nm for 45 min, the intensity of the *trans* band decreased, and *cis* band increased (green line). This confirms the occurrence of *cis*/*trans* isomerization of the azobenzene moiety into SBA-15.

The effect of azobenzene *cis*/*trans* isomerization on the catalytic activity of SBA-15-N-CoL_1_ was investigated by performing the oxidation reaction of cyclohexane with fresh catalyst and UV irradiated catalyst under the optimized conditions. Actually, in the first step the catalyst (100 mg) was first dispersed in acetonitrile (5 mL), then the mixture was exposed to UV light 365 nm for 45 minutes to induce the *trans*-to-*cis* isomerization of the azobenzene groups. In the second step, cyclohexane (1.0 mL, 10 mmol), chlorobenzene (1.0 mL, 10 mmol) as an internal standard, and *m*-CPBA (2.50 mg, 15 mmol), were added to the catalyst mixture. In the last step, the mixture obtained was poured into a sealed autoclave and heated to 60 °C for 2–8 h. The obtained results (Table 6) revealed that UV irradiation was clearly improved the catalytic activity of SBA-15-N-CoL_1_. For example, the cyclohexane conversion and KA oil selectivity were increased from 86%/85% before the UV irradiation to 93%/92% after UV irradiation. The high cyclohexane conversion and KA oil selectivity achieved simultaneously over the SBA-15-N-CoL catalysts can be attributed to key features of the photoactive *cis*-azobenzene complex. Compared to the *trans* isomer, simulations using density functional theory (DFT) showed the *cis* conformation offers more accessible active sites for substrate oxidation.^85,86^ Molecular dynamics simulations further explain that the flexible azobenzene ligands in the *cis* form allow for ideal substrate orientation within the porous framework.^87^ Additionally, two DFT investigations indicate the photoinduced *cis* isomer has a narrower HOMO–LUMO gap than *trans*-azobenzene, consistent with its higher activity in oxidizing cyclohexane.^88^ These effects are complemented by the flexible *cis* structure enabling dynamic accommodation and orientation of reactant/product molecules, as revealed through experimental kinetic isotope effect measurements.^89^ The synergistic impact of factors such as the accessible active sites, dynamic substrate positioning, and electronic structure modulation provided by the light-responsive *cis* complex, helps account for the high conversion and selectivity achieved under mild conditions. This new class of photoactive heterogeneous catalysts extends opportunities for remote performance optimization through photoisomerization.^90^

**Table 6:** Effect of *cis*/*trans* isomerization on the catalytic activity of SBA-15-N-CoL. Cyclohexane: 1.0 mL, chlorobenzene: 1.0 mL, catalyst: 100 mg of SBA-15-N-CoL_1_, oxidant: 2.50 g of *m*-CPBA, solvent: 5 mL of acetonitrile, *T*: 60 °C, UV irradiation: 365 nm for 45 min

Time (h)	Before UV irradiation		After UV irradiation	
	CAXN conversion (%)	KA oil selectivity (%)	CAXN conversion (%)	KA oil selectivity (%)
2	25	23	40	43
4	55	54	67	68
6	79	78	84	85
8	86	85	93	92

The kinetics of the reaction were also studied to determine the catalyst performance. Under the optimized conditions (cyclohexane 1.0 mL (10 mmol), chlorobenzene 1.0 mL, SBA-15-N-CoL_1_ 100 mg, *m*-CPBA 2.50 g as an oxidant, acetonitrile 5 mL as a solvent, 60 °C, 6 h), the molar consumption rate of cyclohexane and generation rate of KA oil over SBA-15-N-CoL_1_ were determined. The molar consumption of cyclohexane was calculated using the equation:
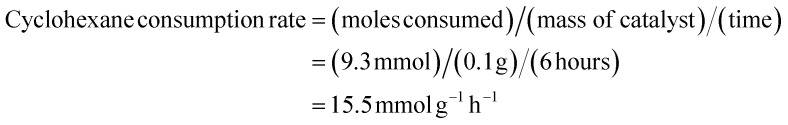


The generation rate of KA oil was calculated using the equation below:
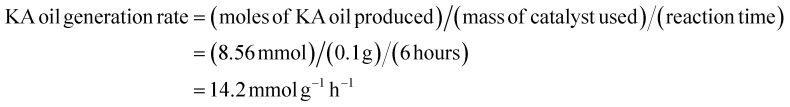


These kinetic parameters of the SBA-15-N-CoL_1_ catalyst confirm its high performance for cyclohexane oxidation.

### 3.3. Catalyst reuse and stability

**Fig. 21:**
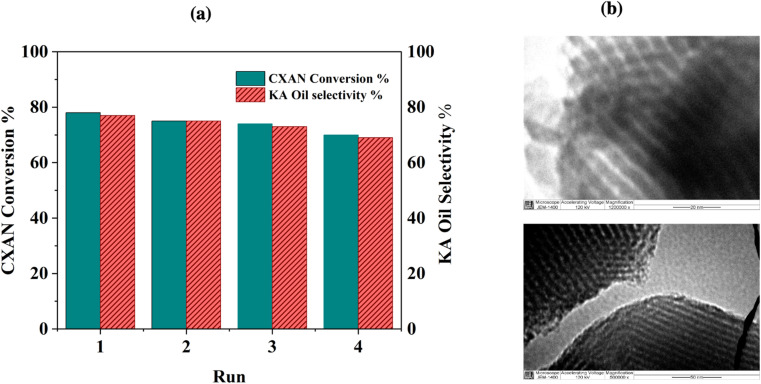
SBA-15-N-CoL_1_ recycling results (a), and TEM images of the spent catalyst (b).

The recyclability of SBA-15-N-CoL_1_ was evaluated through consecutive reaction cycles using the optimized condition. After each run, the catalyst was isolated *via* filtration, washed with chloroform, and dried overnight at 70 °C. The obtained are presented in Fig. 21a. Notably, after 4 consecutive runs, the catalyst maintained high catalytic activity, exhibiting only minor decreases in the conversion and selectivity. Moreover, the nanostructure of the recycled catalyst after the 4 runs was analyzed by TEM and ICP-MS. The characterization outcomes indicated that the structure of the recovered catalyst remained relatively unchanged after 4 cycles, as evidenced by TEM images (Fig. 21b) and ICP-MS results (1.08 wt%).

### 3.4 Proposed mechanism

**Scheme 4:**
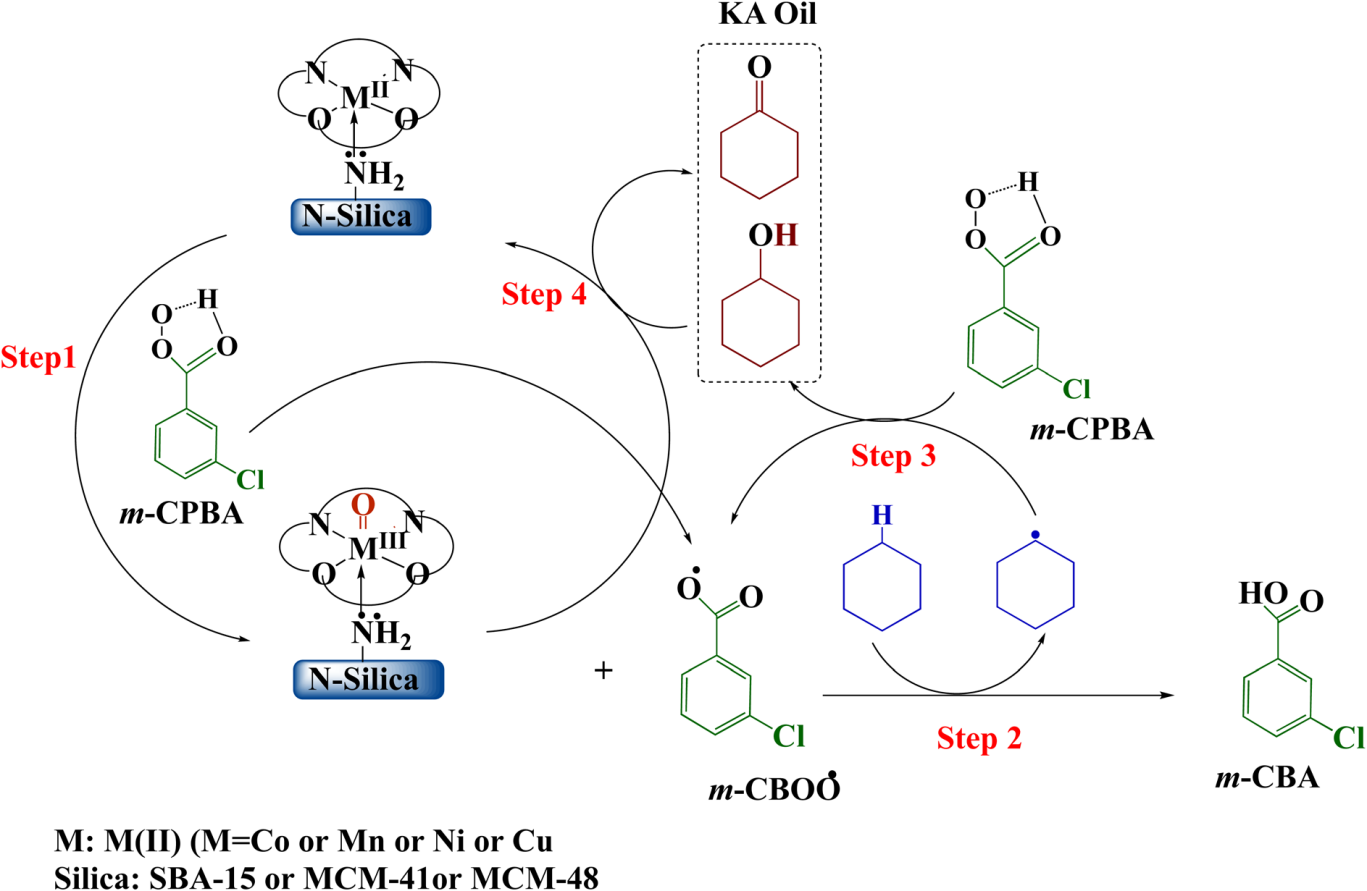
Mechanism of oxidation of cyclohexane over Silica-N-ML_1_ (Silica: SBA-15, MCM-41, MCM-48; M: Co, Mn, Ni, and Cu; L: salphen-azobenzene).

*Meta*-chloroperoxybenzoic acid (*m*-CPBA) is a widely used and effective organic peroxide that serves as a strong oxidizing agent for many different organic reactions. *m*-CPBA has been effectively used to activate the typically unreactive C–H bonds found in hydrocarbons.^91^ The oxidation of C–H bonds is one of the most compelling challenges in organic chemistry. Compared to other common oxidants such as H_2_O_2_ and TBHP, *m*-CPBA exhibits greater stability and selectivity.^92^ These properties are highly advantageous for organic syntheses. *m*-CPBA can also form specialized intermediates when used in conjunction with auxiliary reagents, and these intermediates display enhanced reactivity. Moreover, *m*-CPBA is straightforward to handle as a terminal oxidizing agent.^93,94^ However, in some cases *m*-CPBA may non-selectively generate a variety of radical species. Therefore, a catalyst is needed to activate the O–O bond in a targeted manner and control the reaction pathway.^95^ The catalyst ensures oxidation occurs suitably while suppressing unwanted side and secondary reactions.^96^ Based on prior theoretical studies of transition metal-catalyzed hydrocarbon oxidation and our experimental results, the following mechanism is proposed over Silica-N-ML catalyst (Scheme 4).^97–99^ Through O–O bond homolysis of *m*-CPBA, facilitated by Silica-N-ML active sites, M-oxo (M=O) species and *m*-CBOO˙ radicals are generated (Scheme 4, step 1). The *m*-CBOO˙ acts as cyclohexane (CXAN) C–H bond abstracting agents, producing CXA˙ radicals and *m*-CBA (Scheme 4, step 2). CXA˙ then reacts with additional *m*-CPBA, regenerating *m*-CBOO˙ while forming CXAOL (Scheme 4, step 3). Some CXAOL further undergoes oxidation at Silica-N-ML, yielding CXON (Scheme 4, step 4).

## 4. Conclusion

New and efficient photochromic heterogeneous nanocatalysts were successfully synthesized by immobilizing metallosalphen-azobenzene complexes onto different mesoporous silica surface. A salphen-azobenzene H_2_L_1_ derivative was firs synthetized and complexed with four different transition metals (M: Mn, Co, Ni and Cu). The structure of the obtained complexes [ML_1_] was confirmed by NMR, IR and elemental analysis and PXRD. Then [ML_1_] complexes were incorporated into three different pre-prepared amino-functionalized mesoporous silica (N-Silica: SBA-15-N, MCM-41-N, and MCM-48-N) *via* coordination bonds. The twelve prepared catalysts Silica-N-ML_1_ were fully characterized by different techniques such as FT-IR, SEM, TEM, XRD, ICP-MS, DR UV-Vis and N_2_ physisorption. The obtained results confirmed the successful grafting of APTES and immobilization of [ML_1_] complexes onto silica surface, with the preservation of the silica mesoporosity and nanostructure order. Results revealed also the presence of *trans* configuration of the azobenzene group as the major isomer in Silica-N-ML_1_ materials, which was easily transformed to *cis* isomer upon UV irradiation. The catalytic activity of the prepared nanocatalyst (Silica-N-ML_1_) was evaluated in the oxidation reaction of cyclohexane to produce KA oil. Different parameters were investigated to determine the optimized conditions, such as type of oxidant, type of silica, type of metal, catalyst dose, reaction time, temperature, and UV light. The best results were obtained with 100 mg of SBA-15-N-CoL_1_, using *m*-CPBA as oxidant, at 60 °C, for 6 h, and under UV light. A superior catalytic activity was observed for the *cis* conformation under UV light, achieving 93% conversion and 92% selectivity toward KA oil. Moreover, the SBA-15-N-CoL_1_ nanocatalyst exhibited a good catalytic activity performance and high stability in four consecutive cycles. Leaching measurement using ICP-MS and TEM images of the spent catalyst confirmed an excellent stability of this photochromic nanocatalyst.

## Data availability

Data available upon request.

## Conflicts of interest

The authors declare that they have no known competing financial interests or personal relationships that could have appeared to influence the work reported in this paper.
